# Real Time and Spatiotemporal Quantification of pH and H_2_O_2_ Imbalances with a Multiplex Surface-Enhanced Raman Spectroscopy Nanosensor

**DOI:** 10.1021/acsmaterialsau.2c00069

**Published:** 2023-02-15

**Authors:** Can Xiao, Victor Izquierdo-Roca, Pilar Rivera-Gil

**Affiliations:** † Department of Medicine and Life Sciences, Universitat Pompeu Fabra, Carrer Doctor Aiguader 88, 08003 Barcelona, Spain; ‡ Catalonia Institute for Energy Research (IREC), Jardins de les Dones de Negre 1, 08930 Sant Adrià del Besòs-Barcelona, Spain

**Keywords:** colloidal plasmonic nanocapsules, surface-enhanced Raman scattering, aromatic boronic acid sensors, pH and H_2_O_2_ biosensing, multiplex biosensors, cell homeostasis

## Abstract

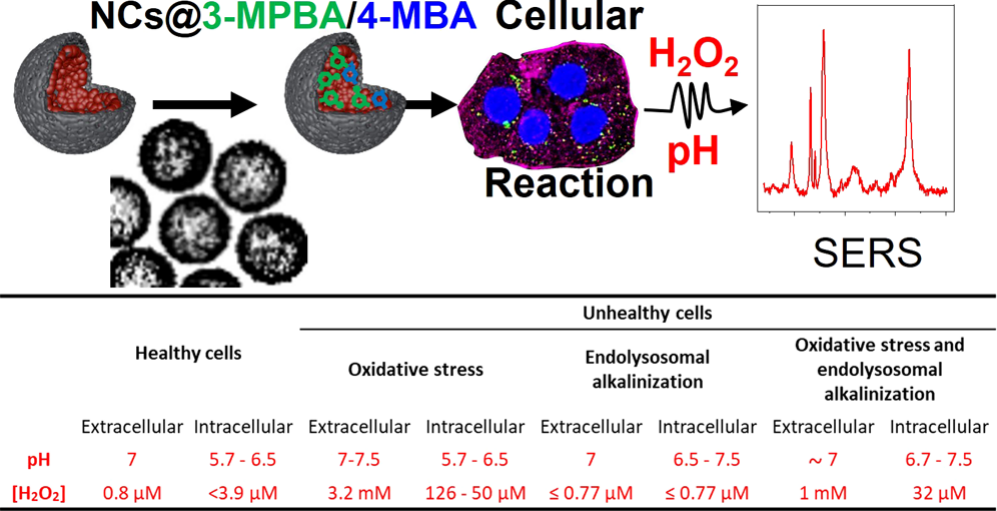

Oxidative stress is involved in many aging-related pathological disorders and is the result of defective cellular management of redox reactions. Particularly, hydrogen peroxide (H_2_O_2_), is a major byproduct and a common oxidative stress biomarker. Monitoring its dynamics and a direct correlation to diseases remains a challenge due to the complexity of redox reactions. Sensitivity and specificity are major drawbacks for H_2_O_2_ sensors regardless of their readout. Luminiscent boronate-based probes such as 3-mercaptophenylboronic acid (3-MPBA) are emerging as the most effective quantitation tool due to their specificity and sensitivity. Problems associated with these probes are limited intracellular sensing, water solubility, selectivity, and quenching. We have synthesized a boronate-based nanosensor with a surface-enhanced Raman spectroscopy (SERS) readout to solve these challenges. Furthermore, we found out that environmental pH gradients, as found in biological samples, affect the sensitivity of boronate-based sensors. When the sensor is in an alkaline environment, the oxidation of 3-MPBA by H_2_O_2_ is more favored than in an acidic environment. This leads to different H_2_O_2_ measurements depending on pH. To solve this issue, we synthesized a multiplex nanosensor capable of concomitantly quantifying pH and H_2_O_2_. Our nanosensor first measures the local pH and based on this value, provides the amount of H_2_O_2_. It seems that this pH-dependent sensitivity effect applies to all boronic acid based probes. We tested the multiplexing ability by quantitatively measuring intra- and extracellular pH and H_2_O_2_ dynamics under physiological and pathological conditions on healthy cells and cells in which H^+^ and/or H_2_O_2_ homeostasis has been altered.

## Introduction

The presence and progression of certain diseases are associated with changes of biomolecules.^1^ There are close interrelations between disease markers which can affect clinical outcome. To understand cellular mechanisms in the healthy state and disease progression, multiplex analysis of biomolecules in a complex mixture is essential.^2,3^ Compared with classical bioanalytical strategies, in which each analyte is individually determined, multiplex technologies permit simultaneous measurements of multiple analytes in a single run of the assay within a small sample.^4^ Multiplex sensing is a rapid and accurate diagnostic method, which also provides significantly more information about the health state of an individual.^5^

Redox regulation (control and signaling) are fundamental physiological reactions in living cells. Oxidative stress is related to abnormal management of redox chemistry and altered homeostasis of reactive species.^6^ It is a common factor involved in aging^7^ and in many pathological conditions such as some types of cancer, cardiovascular and neurodegenerative diseases,^8^ and diabetes.^9,10^ Some of them have even been named “redox diseases”. There is a complex reactive species interactome network involving reactive oxygen, nitrogen, and sulfur species (ROS, RNS, RSS).^11^ The ROS hydrogen peroxide (H_2_O_2_) is one of the most important transcription independent signal molecules,^12^ serving as a key metabolite in redox sensing, signaling, and redox regulation, because of its unique chemistry properties (long lifetime and uncharged nature) allowing transportation and remote signaling.^13^ Cellular effects are initiated under permeation of H_2_O_2_ through cells and tissues. Average intracellular H_2_O_2_ physiological concentration in mammalian cells likely ranges from 1 to 700 nM. Stress and adaptive stress responses, even inflammatory responses and cell death, occur at higher H_2_O_2_ concentrations.^14^ For better understanding of redox reactions and for better control of redox therapeutics, facile measurement of the intracellular concentration of hydrogen peroxide has been a focused interest and a long-standing challenge.

Fluorescent probes are suitable to record the spatiotemporal distribution of H_2_O_2_.^15^ Classical chemical assays for H_2_O_2_ determination are horseradish peroxidase (HRP)-dependent probes but are limited to extracellularly available H_2_O_2_.^15^ There are plenty of H_2_O_2_ reporters for biological sensing; however their major drawback is rendering them water soluble and maintaining the specificity for H_2_O_2_.^16^ More sophisticated sensors are genetically encoded fluorescent indicators, like HyPer probe^17^ and roGFP-orp1,^18^ which are designed to be used for intracellular H_2_O_2_. Despite the common fluorescence limitations, e.g., complicated synthesis of probes and photobleaching issues, they have their specific limitations. HyPer probes are pH sensitive, and the manipulation is complicated for roGFP-orp1, and *in situ* quantification is complicated to realize, which hampers their popularity.^15^ Boronate-based fluorescent probes are emerging as one of the most effective sensors for redox biology.^15,16,19−22^ Considering that these molecular probes are designed to quantify redox species in cellular structures and that each of them exhibit different pH values ranging from 3 to 8, the environmental pH value could have a clear impact on redox sensing and quantification, thus resulting in misleading conclusions. To the best of our knowledge, none of these reports consider the effect of the pH in boronate-based sensor’s response.

Surface-enhanced Raman spectroscopy (SERS) retains the rich chemical and structural information provided by Raman spectroscopy but overcomes its inherent limitation to the investigation of low amounts of material, especially when interparticle hot-spots occur.^23^ This provides a nondestructive and sensitive tool to investigate chemical modifications of the probe molecule onto the platform since the analyte recognition can induce characteristic spectral changes. Nanosphere,^24,25^ core–satellite,^26^ and core–shell^27^ nanoparticles based on gold have been previously reported for H_2_O_2_ SERS sensing. The Raman probes used for *in vitro* H_2_O_2_ SERS sensing are mainly boronate molecules with high Raman cross-section: 3-mercaptophenylboronic acid (3-MPBA),^24^ 4-MPBA,^26^ and others.^25^ Rezende et al.^15^ reported that the common limitation of boronate based probes for H_2_O_2_ detection is the interference coming from some peroxynitrite species, such as ONOO^–^; however other works like that of Gu et al.^24^ reported 3-MPBA modified nanoparticles, similar to ours, showing no interference with other ROS and RNS. Remarkably, all referred nanoparticles carried the Raman probes in the outer side of the nanostructure, i.e., at the interface with the media. Biomolecules can come in close contact and further be adsorbed on a metallic surface when nanoparticles are exposed to biological media.^28,29^ The presence of biomolecules (e.g., glutathione) can replace or remove the Raman probes from the metallic surface since they are not well protected.^30^ Biomolecule close contact with the metallic surface induces detectable Raman signals that can interfere with the signal of the Raman probes. This has important limitations hampering the sensitivity and reliability of the quantification, thereby compromising their biomedical sensing applications. Besides, the reported nanoparticles are in a size range below 100 nm; thus isolated nanoparticles are not visible with a Raman microscope. Uncontrollable agglomeration and aggregation occur in biological and physiological media, which can induce strong heterogeneous SERS response.^31^ Moreover, none of the articles discussed the pH effect on aromatic boronate oxidation for H_2_O_2_ sensing. Considering that the sensors are internalized in cellular vesicles with different pH values, identifying the location and the local pH are essential for H_2_O_2_ sensing.

Lysosomal H_2_O_2_ reacts with labile iron forming hydroxyl radicals, which may cause lysosomal rupture and further proapoptotic cascade.^32^ And the accumulation of peroxidized lipids and proteins in lysosomes of brain cells is one of the known factors in Alzheimer’s disease.^22^ To complete the intracellular H_2_O_2_ profile, apart from previous studies on mitochondrial and cytosolic H_2_O_2_,^17,33^ it is important to understand lysosomal H_2_O_2_. Another aspect is that concentration gradients exist both from extracellular to intracellular and between subcellular compartments.^13^ Previous estimations suggested that extracellular H_2_O_2_ is around 10-fold^34^ or 650-fold^35^ higher than intracellular concentration, due to intracellular H_2_O_2_ metabolism,^36^ varying with cell type and locations inside cells and various parameters.^13,37^ By building a compartmental model to estimate the gradients between extracellular and intracellular H_2_O_2_, the intracellular H_2_O_2_ concentration and cellular responses can be potentially estimated by simply observing extracellular H_2_O_2_ perturbations.^35^ To address this need, sensors which can be used for both extracellular and intracellular monitoring are required.

In this study, we wanted to design and validate SERS for multiplexing since we have already demonstrated the uniqueness of this readout to unequivocally trace biomarkers in monovalent sensors.^38−40^ The chemical biology of the ROS species, H_2_O_2_, is very complex and there is a gap between the molecular mechanisms of H_2_O_2_ and diseases like aging or cancer. Considering that boronate-based probes are the state of the art in H_2_O_2_ sensing and the fact that there is no report showing the impact of microenvironmental pH on their sensing, we selected the boronate probe, 3-MPBA, and 4-mercaptobenzoic acid (4-MBA) for H_2_O_2_ and pH concomitant quantification, respectively. We took advantage of the fact that our plasmonic nanostructure produces strong and homogeneous SERS response and allows for single-nanocapsule analysis taking advantage of interparticle hot-spots concentrated in their inner shell. Moreover, the silica shell offers intrinsic resistance against aggregation and prevents physicochemical interaction between the gold nanoparticles and the proteins from biological media, thus effectively repelling protein fouling. All this ensures that the signal of our nanosensor is stable and reproducible. We present a biocompatible, noninvaisve multiplex sensor for intracellular and extracellular H_2_O_2_ and pH quantification in real time and with spatiotemporal resolution.

## Materials and Methods

### Materials and Reagents

3-MPBA, 4-MBA, 3-mercaptophenol (3-MP), 2,2′-azobis(2-methylpropionamidine) dihydrochloride (AIBA), polyvinylpyrrolidone (PVP, MW 10000), styrene, poly(sodium styrenesulfonate) (PSS, MW 70000), poly(allylamine hydrochloride) (PAH, MW 50000), tetrakis(hydroxymethyl) phosphonium chloride solution (THPC), gold(III) chloride trihydrate, ammonia solution, tetraethyl orthosilicate (TEOS), phosphoric acid, sodium phosphate monobasic, sodium phosphate dibasic, hydrogen peroxide solution and menadione were purchased from Sigma-Aldrich. Sodium hydroxide, LysoTracker, Cellmask, and Mitotracker were purchased from ThermoFisher. Bafilomycin A1 was purchased from ChemCruz, and chloroform was purchased from Scharlau. All the chemicals were used without further purification.

Polystyrene (PS) beads were synthesized as previous reported.^41^ Polymerization was carried out with AIBA as an initiator. Styrene was added to PVP and AIBA mixture at 70 °C. The reaction was kept at 70 °C for 24 h.

PSS solution and PAH solution (1 mg/mL containing 0.5 M NaCl) were prepared freshly before use. 100 mM phosphate buffer with pH ranging from 4 to 9 was prepared with phosphoric acid, sodium phosphate monobasic, and sodium phosphate dibasic. By adding series concentrations of H_2_O_2_ solution into phosphate buffer (0.5% (v/v)), H_2_O_2_ concentration from 10^–2^ M to 10^–8^ M under the full range of pH was obtained. pH was measured again and confirmed to be maintained after H_2_O_2_ addition.

### Nanocapsule Synthesis

Nanocapsules (NCs) were produced with the method reported.^40^ Briefly, we use a template of polystyrene (PS) beads that was decorated with gold nanoparticles by using a layer-by-layer (LbL) assembly protocol. Negatively charged PSS and positively charged PAH were alternately deposited onto PS beads of 450 nm diameter to form a final dense external layer of PAH. Consecutively, significant excesses of negatively charged 2–3 nm diameter of Au nanoparticles (Au-seeds) were added and left to adhere via electrostatic interaction. The formed structures, PS@Au-seeds, were then extensively washed to remove the unbound nanoparticles. Thereafter, PS@Au-seeds were coated with a polyvinylpyrrolidone (PVP) layer and covered with a silica shell. Hollow silica capsules containing Au-seeds were obtained by dissolving the PS cores with an ethanol/chloroform mixture. To increase the plasmonic efficiency of the nanostructure, Au-seeds inside the NCs were grown by *in situ* seed catalyzed reduction of gold ions with formaldehyde.

For the internalization studies performed with the confocal microscope, we used a polystyrene template labeled with a fluorophore (Ex/Em, 576/596) bought from Ikerlat. Then we synthesized the nanocapsules following the same protocol, but we did not remove the template at the end of the synthesis.

### Morphological Characterization

The morphology of the NCs synthesized have been examined by using a JEOL JEM 1010 transmission electron microscope (TEM) operating at an acceleration voltage of 80 kV with a tungsten filament. The absorption spectrum of each synthetic intermediate has been analyzed with an UV–vis spectrometer (GE Healthcare Ultrospec 2100 pro). Dynamic light scattering (DLS) and zeta potential analysis were performed with Zetasizer Nano ZS (Malvern Instruments, UK) which is capable of both particle size analysis and zeta-potential measurement.

### SERS Sensor Preparation

Mixed self-assembled monolayer (SAM) methodology was used for the modification of NCs. Nanosensors were obtained by saturating the gold surface with thiolated aromatic molecules (3-MPBA, 3-MP, and 4-MBA). 3-MPBA, 3-MP, and 4-MBA feedstock solutions were prepared with a concentration of 5 mM in ethanol. NCs were mixed and incubated with feedstock solutions for at least 3 h, followed by centrifugation to remove the excess molecules. Centrifugation (5000 rcf, 2 min) was repeated 4 times. After each centrifugation, NCs were resuspended into ethanol.

### SERS Measurements

The laser was focused onto the samples with a 60× (NA 1.00) water immersion objective, providing a laser spot diameter of approximate 1 μm. The inelastic radiation was collected with a Renishaw’s inVia Qontor Raman system equipped with a confocal optical microscope, a grating of 1200 l·mm^–1^, a NIR laser (785 nm), and a Peltier cooled CCD array detector. Samples were studied with Windows-based Raman Environment (Wire) software.

Glass bottom dishes (IBIDI) were used for Raman measurements. For non-cell samples, modified NCs were suspended into phosphate buffer or cell growth medium with a concentration of 0.018 pmol/L (calculated by number of NCs). NCs were incubated with H_2_O_2_ solution for 30 min before Raman measurements. Integration time was set to 11 s with power at the sample of 5 mW. Laser power of 5 mW and exposure time 15 s were used to excite intracellular nanosensors. Living cells were incubated in growth media when collecting Raman signals.

The preprocessing steps were done with Spyder (anaconda3). Spectra baselines were subtracted by asymmetric least-squares smoothing algorithm in order to eliminate the auto fluorescence background.^42^

An analysis of variance of Raman spectra has been performed to identify the wavenumber range with relevant spectral variations under the different pH and H_2_O_2_ conditions. The quantification of the variance of the spectra has been carried out by calculating the standard deviation (pixel by pixel) between different spectra acquired under different conditions. The relevant spectrum variance threshold has been determined using the criterion that only regions with spectrum variance greater than 1 order of magnitude of noise deviation (calculated in the region without detectable peaks, 1620–1680 cm^–1^) are significant.

### Cell Culture and Viability Assay

HT29 cells (colon cancer cells) were cultured in Dulbecco’s Modified Eagle Medium/Nutrient Mixture F-12 (DMEM/F12, Thermofisher) supplemented with 10% fetal bovine serum (FBS), 1% l-glutamine, and 1% penicillin–streptomycin.

Cell viability assay was conducted with *In Vitro* Toxicology Assay Kit (Resazurin based). 20000 cells per well HT29 cells were seeded in a 96-well plate in triplicate in 100 μL of growth medium. After cell attachment and 70% confluence, cells were treated with NCs with concentrations from 0.018 to 2.3 pmol/L. After 24 h of incubation, a solution of 10% resazurin in cell growth media was added to each well at a final volume of 100 μL/well. Then, cells were placed for 3 h in the incubator to metabolize the resazurin (nonfluorescent compound) into resorufin (fluorescent compound). The 96-well plate was read by fluorescence measurement, 560 and 580 nm for excitation and emission, respectively, using a fluorescence spectrophotometer (Agilent Technologies). Fluorescence intensities of treated samples were normalized to the untreated control (cells without NCs treatment). Data was plotted with GraphPad Prism6.

### *In Vitro* Experiments

HT29 were grown onto glass bottom dishes (IBIDI). After sufficient cell attachment, 0.072 pmol/L modified NCs were incubated with HT29 for 24 h. Living cells were incubated in cell growth medium for Raman measurements. The internalization of NCs by HT29 after 24 h was verified with a confocal laser scanning microscope (CLSM) (Leica TCS SP5 AOBS (inverted)). For H_2_O_2_ treated HT29, cells were incubated with 0.5 to 10 mM H_2_O_2_ in cell growth media for 30 min before Raman and CLSM analysis. Intracellular H_2_O_2_ was checked by CLSM with Premo Cellular Hydrogen Peroxide Sensor. For bafilomycin A1 stimulation, HT29 cells were incubated with 500 nM bafilomycin A1 in cell growth media for 2 h. pH changes inside lysosomes were verified by CLSM with LysoTracker Green.

## Results and Discussion

### Multiplex Nanosensor Synthesis and Characterization

**Figure 1:**
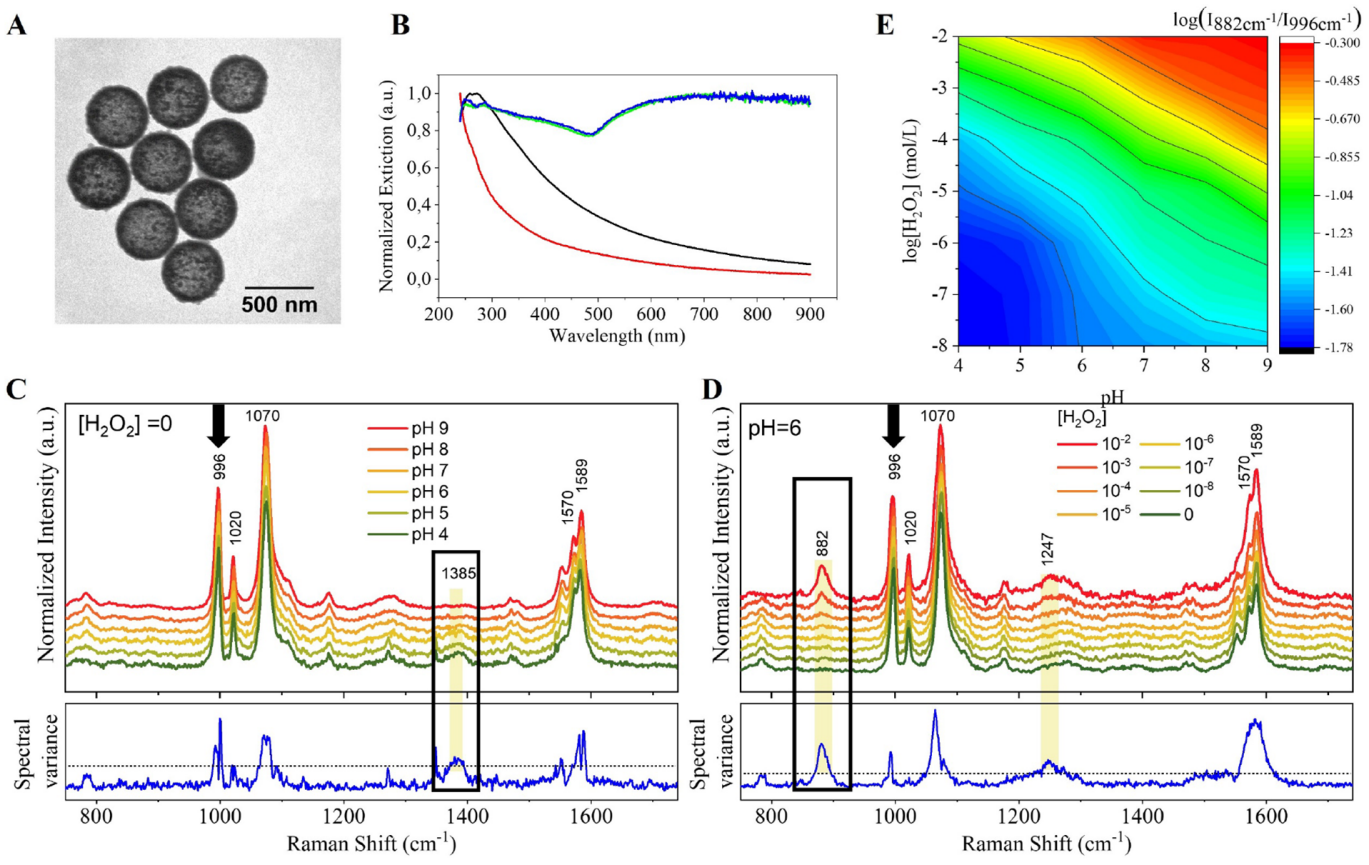
Synthesis, characterization, and NCs@3-MPBA&4-MBA’s responsiveness to H_2_O_2_ and pH dynamics. (A) TEM image of AuNPs@SiO_2_ NCs. The size of the NCs was approximately 450 nm. (B) UV–visible extinction spectra of the different steps during their synthesis. Black line: PS beads; Red line: Au seeds@SiO_2_ NCs; Green line: AuNPs@SiO_2_ NCs; Blue line: hollow NCs@3-MPBA. (C–E) SERS spectra and spectra variance of NCs@3-MPBA&4-MBA: (C) at different pH in the absence of H_2_O_2_, and (D) at different H_2_O_2_ concentration and fixed pH 6. Yellow-shadowed regions indicate the spectral ranges with higher variance and the black arrows point to the selected reference (insensitive) peak. (E) 3D matrix showing the correlation between log(*I*_882_/*I*_996_) and H_2_O_2_ (10^–2^ M to 10^–8^ M) under different pH values (4–9) of NCs@3-MPBA&4-MBA. Each point is the average of five probes.

We have synthesized a complex nanostructure composed of hollow polymeric silica NCs with a high density of plasmonic gold nanoparticles placed on the inner surface of the NCs following a previously established protocol.^40^Figure 1A shows the TEM characterization of the plasmonic NCs with an average diameter of 450 nm, disclosing nanoparticle homogeneity and the porosity of the silica shell that allows the diffusion of small molecules^38^ such as H_2_O_2_ and ions like H^+^. More TEM images of the different synthetic intermediates generated during NCs synthesis are available in the Supporting Information (SI) (SI section 1, Figure SI-1A–C). DLS confirms the homogeneity of the NCs in suspension showing an average hydrodynamic size of 467.6 nm (SI section 1, Figure SI-1D) and zeta potential value of −36.7 mV (SI section 1, Figure SI-1E) due to the presence of deprotonated silanol groups in the silica shell. Figure 1B presents the normalized extinction spectra of the NCs during the different steps of the synthesis. The black line shows the overall spectrum of the colloidal template (polystyrene, PS, beads) displaying a large scattering background with a well-defined band centered at approximately 290 nm and a long tail at longer wavelengths. The red line represents the spectrum of hollow NCs, functionalized with Au seeds before growing the plasmonic nanostructure. It proves that PS was correctly removed and that we obtained hollow NCs. At this point of the synthesis, the NCs have Au seeds (2–3 nm), and therefore, there is no characteristic localized surface plasmon resonances (LSPRs). The green line corresponds to the spectrum of the NCs after growing the plasmonic nanostructure. The dominant contribution shifts to higher wavelengths and broadens, indicating the significant formation of gold nanoparticle agglomerates and plasmon coupling. This hybrid material acts as a robust nanocarrier of large ensembles of interparticle hot spots concentrated in their internal surface. This provides high SERS activity via interparticle coupling and a highly averaged plasmonic response that ensures great homogeneity within capsule-to-capsule Raman signal enhancement. To obtain the colloidal multiplex nanosensor, we saturated the gold surface with thiolated aromatic probes with a high Raman cross section, i.e., 3-MPBA and 4-MBA for H_2_O_2_ ROS species and pH, respectively (NCs@3-MPBA&4-MBA). The functionalization of the NC surface occurs via a strong covalent gold–thiol bond. The blue line in Figure 1B confirms no changes in the NCs’ extinction spectra when the Raman probes were adsorbed onto the metallic gold surface. We did observe spectral fingerprint differences between the free and the NC-adsorbed Raman probes (Figure SI-1F) because of the surface selection rules and the surface enhancement due to the media interaction and the resonance coupling occurring when the Raman probe is adsorbed onto the NCs’ metallic surface.

We selected 3-MPBA as a H_2_O_2_ sensor molecule because it can be oxidized into 3-MP in the presence of H_2_O_2_ showing new SERS characteristic bands of 3-MP^24^ (Figure SI-1G). A comparison to other H_2_O_2_ sensors is presented in Table SI-1 (SI section 1). Then, we identified which bands are sensitive to H_2_O_2_. We performed a statistical analysis of the spectral variation based on standard deviations to detect significant spectral variation of 3-MPBA modified NCs (NCs@3-MPBA) dependent on H_2_O_2_ (SI section 1, Figure SI-2A). Figure SI-2B (SI section 1) shows the assignment of the main 3-MPBA peaks and their evolution in NCs@3-MPBA SERS spectra with and without H_2_O_2_. We considered spectral variations higher than 25% as sensitive to H_2_O_2_. We identify the peak sensitive to H_2_O_2_ concentration at 882 cm^–1^ corresponding to the oxidation of 3-MPBA to 3-MP (Figure SI-1G), consistent with previous studies.^24^ Moreover, the oxidation of 3-MPBA did not change significantly the molecular orientation of the conjugated Raman probe since we did not monitor a large set of different perturbations on the SERS spectra^43^ (SI section 1, Figure SI-1F). We also conclude that the peak at 996 cm^–1^ is not affected by the oxidation of the molecule, thus being insensitive to H_2_O_2_. This invariance of the intensity allows us to use this contribution as a reference band for ratiometric analysis to minimize the impact of external parameters such as NC batch-to-batch variability or different cellular loading. Figure SI-3A–C (SI section 1) shows the relation between intensity ratio of 882 and 996 cm^–1^ and the concentration of H_2_O_2_ of the NCs@3-MPBA over time dispersed in phosphate buffered saline (pH 7, high ionic strength) (SI section 1, Figure SI-3A) vs. cell growth media (pH ≈ 7.5, high ionic strength and high content of biomolecules; SI section 1, Figure SI-3B). We finally plotted the calibration curves in both media. Both curves show the same trend, proving the validity of our sensor for the ROS species H_2_O_2_ quantification in biological environments (SI section 1, Figure SI-3C).

4-MBA has been previously used for pH sensing with SERS,^39,44^ since the ratiometric intensity signal of the peaks at 1385 cm^–1^ associated with COOH groups^45^ and the insensitive peak at 996 cm^–1^ can be calibrated for pH sensing. Figure SI-4 (SI section 1) confirms that the SERS response of 4-MBA was not altered by the presence or absence of H_2_O_2_ and remained constant, therefore confirming no crosstalk between the signals and ensuring specific pH quantification.

Once we confirmed the sensitivity of both Raman probes for pH and H_2_O_2_ sensing, we synthesized the multiplex nanosensor composed of NCs containing 3-MPBA and 4-MBA (NCs@3-MPBA&4-MBA) following the same procedure as described for the individual sensors. This was not straightforward as both Raman probes compete for the same plasmonic surface area, but they occupy different volumes, and they exhibit different Raman cross sections. If randomly added to the NCs, the signals could interfere with each other and provide erratic results. Therefore, we considered it important to homogenize signal intensities of both Raman probes before sensing. To do this, we estimated the relative amount of each Raman probe on the plasmonic surface. First, we selected and compared two characteristic bands (like 996 and 1075 cm^–1^) for both analytes exhibiting high intensity and analyte-insensitivity (SI section 1, Figure SI-5). Then we merged the normalized SERS spectra of each probe in a 1:1 ratio (SI section 1, Figure SI-5A), mimicking a situation where both signals are equal, and we calculated the intensity ratio of these two bands. When the ratio is around 1.55 corresponding to 3-MPBA/4-MBA concentration ratio of 15:1 (SI section 1, Figure SI-5B), the signal of both probes is the same (SI section 1, Figure SI-5C). Finally, we tested and confirmed the responsiveness of the multiplexed nanosensor. Within the NCs@3-MPBA&4-MBA, the responsiveness of 3-MPBA and 4-MBA toward both analytes, H_2_O_2_ and pH, was maintained in the presence of each other in terms of intensity and analyte specificity with no crosstalk between the signals (Figure 1C,D). At fixed H_2_O_2_ (0 M, Figure 1C) or pH (pH 6, Figure 1D), the sensitive and insensitive bands used to quantify H_2_O_2_ or pH, respectively, showed the same SERS response dependent on each analyte’s concentration as the individual nanosensors did (NCs@&4-MBA, SI section 1, Figure SI-4; NCs@3-MPBA, SI section 1, Figures SI-2 and SI-3). We can conclude that there is no interference of one analyte into the SERS response of the other analyte’s sensor, thus excluding cross talk. The multiplexed nanosensor is sensitive to pH changes in the physiological range (Figure 1C). We plotted the ratio between the pH sensitive and insensitive bands to obtain the multiplex nanosensor’s calibration curves for pH (*I*_1385_/*I*_996_). They exhibited the typical Henderson–Hasselbalch plot^46^ (SI section 1, Figure SI-6A), which is in agreement with the SERS-based nanosensors.^44^ The multiplexed nanosensor is also sensitive to H_2_O_2_ changes in the physiopathological range and the specificity of the response was maintained toward analyte concentration at a fixed pH (Figure 1D). However, when we measured the multiplex nanosensor’s response toward H_2_O_2_ while varying the pH, we did observe differences in the H_2_O_2_ measurements (Figure 1E and SI section 1, Figure SI-6B showing all spectra). In the next section, we studied in detail the dependency of the multiplex nanosensor and more specifically the boronate-based sensor (3-MPBA) signaling on pH to be able to build the multiplex nanosensor’s calibration curve for H_2_O_2_.

### Influence of Physiological pH Levels on Boronate-Based H_2_O_2_ Sensor Sensitivity

The influence of pH is not always studied when reporting biological sensors. Intracellular pH varies among different cellular compartments. Organelles of endocytic pathways also have different luminal acidity (pH 4.7 to 6.7), while cytosol pH ranges between 7.0 and 7.4, and extracellular pH ranges between 7.3 and 7.6.^47^ Only few reports have demonstrated that pH alters analyte sensing in cells.^39,48−51^ Specifically for redox (patho)physiology, the latest most effective tools use boronate-based sensors like 4-MPBA; however to the best of our knowledge, none of these reports consider the effect of pH in the sensor response.^15,16,19−22^ We have observed that the response is different depending on the environmental pH. Therefore, to accurately determine local H_2_O_2_ concentrations in cells using our multiplex nanosensor, it is necessary to study the influence of pH. We noticed that the H_2_O_2_ signal increases with the alkalinization of the environment in a H_2_O_2_-concentration dependent manner. We observed this phenomenon in the multiplex nanosensor (NCs@3-MPBA&4-MBA, Figure 1E and SI section 1, SI-6B) but also in the individual sensor (NCs@3-MPBA, SI section 2, Figure SI-7). At fixed H_2_O_2_ concentration, the sensitive bands used to quantify H_2_O_2_ increase with the pH. Interestingly, in the absence of H_2_O_2_ (SI section 2, Figure SI-7A, green spectrum), those same bands are not affected by pH, thus confirming no cross talk between the signals and signal specificity. Our results show a pH effect on the H_2_O_2_ quantification. However, this influence is not promoted by the coexistence of both sensors in the same plasmonic surface nor by a pH-mediated cross talk of the H_2_O_2_ signaling bands. Therefore, there must be a different underlying mechanism.

**Figure 2:**
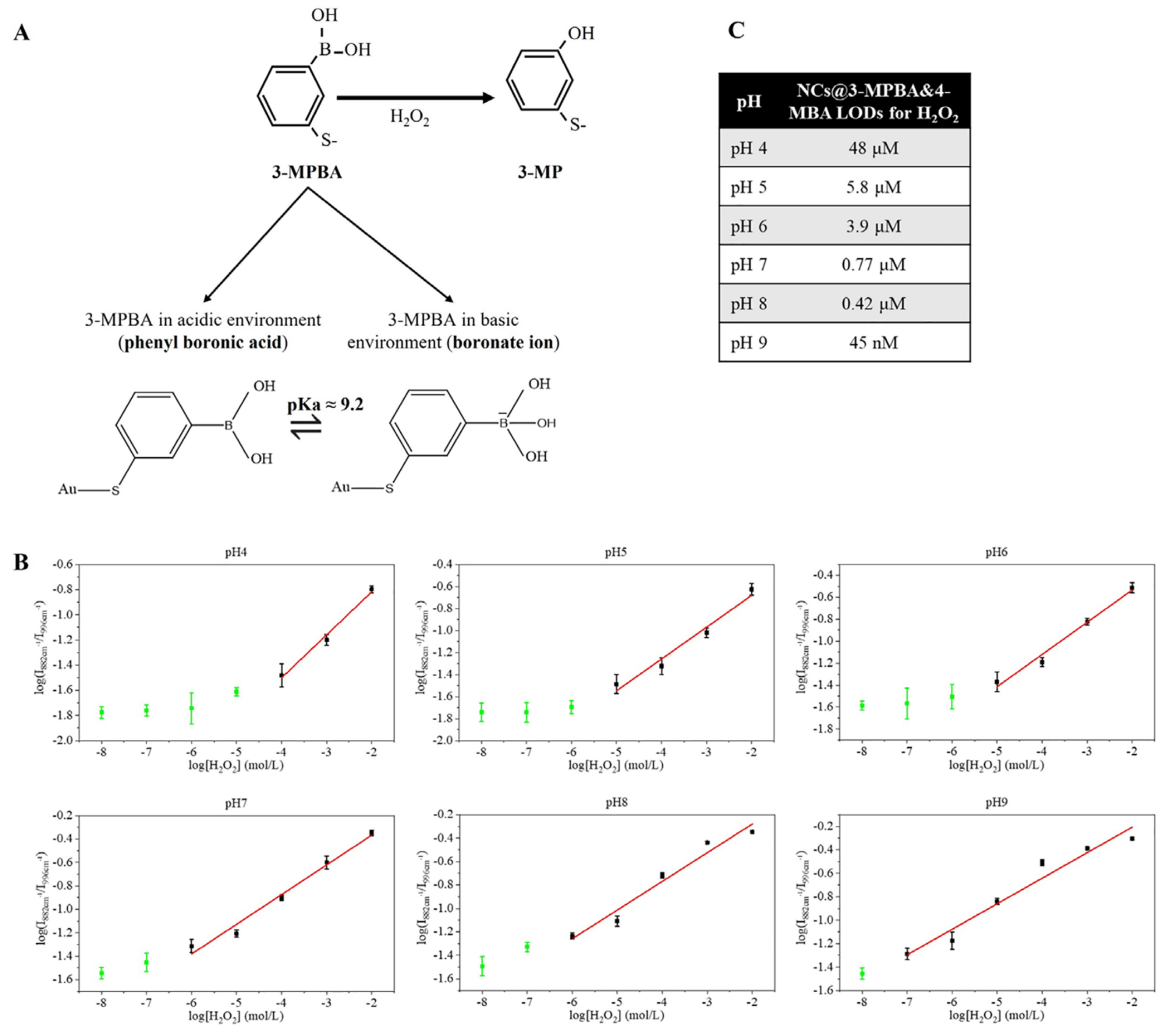
NCs@3-MPBA&4-MBA H_2_O_2_ calibration curves and limits of detection. (A) Scheme of the H_2_O_2_-mediated oxidation of 3-MPBA into 3-MP and the different molecular variants of 3-MPBA depending on the pH, i.e., phenylboronic acid at acidic pH and boronate at alkaline pH. (B) Calibration curves of NCs@3-MPBA&4-MBA for H_2_O_2_ sensing were obtained in phosphate buffer with pH ranging from 4 to 9. Red lines are linear fitting results. Green dots are masked data (data points not included in the linear fitting). Error bars represented the standard deviations of five probes. (C) NCs@3-MPBA&4-MBA LODs for H_2_O_2_ sensing depending on pH. For more information, see SI section 2.

To further elucidate this mechanism that seems to be intrinsic to all boronic acid based sensor probes, we performed a set of experiments on the sensor. Our integrated molecular sensor for H_2_O_2_ is 3-MPBA. The sensing mechanism involves H_2_O_2_ oxidation of 3-MPBA into 3-MP (Figure 2A). We first studied the effect of pH on the SERS spectra of the oxidized form (3-MP) and observed no change (e.g., band shift, intensity ratios, among others) in its vibrational mode between pH 4 and pH 9 (SI section 2, Figure SI-8). This confirms that the pH effect on the sensor’s responses is not related to 3-MP vibrational differences, and therefore, we can ensure that we have no pH crosstalk on the H_2_O_2_ signal and that our nanosensor’s response is specific toward H_2_O_2_ changes. However, why the amounts measured are different depending on pH remains to be elucidated.

Let us have a look now to the molecule 3-MPBA. During its H_2_O_2_-mediated oxidation into 3-MP, the boronate functional group changes into a hydroxyl group because of the rupture of the B–C chemical bond (Figure 2A). The equilibrium constants of the oxidation reaction occurring in our nanosensor vary at specific pH. This can be estimated using the Henderson–Hasselbach equation and the p*K*_a_ of 3-MPBA. The p*K*_a_ measures the Lewis acidity and is an important parameter of biological sensors. The p*K*_a_ of 3-MPBA is around 9.2.^52^ It determines the ratio between the ionic species of 3-MPBA. Below the p*K*_a_, it carries a trigonal boronic acid which changes into a tetragonal boronate ion above the p*K*_a_, thus changing the molecular structure and becoming negatively charged at pH ≥ 9.2 (Figure 2A and SI section 2, Figure SI-9A). Our nanosensor is detecting these changes in the molecule occurring during charge transfer (CT) processes.^53^ We confirmed this by monitoring a band that is sensitive to CT processes (1553 cm^–1^) and confirming that this band varies with the environmental pH values (SI section 2, Figure SI-9B). These results also demonstrate that our nanosensor is highly sensitive and specific as it detects molecular changes of the same molecule.

Under alkaline pH, there is an excess of the negative species and an increased formation of boronate complexation with the three hydroxyl groups, compared to more acidic dispersions. This results in an enhancement of the B–C cleavage sensitivity.^54^ In other words, at basic pH, the H_2_O_2_-mediated oxidation of the sensor is facilitated, and that is why our sensors measure an increase in the H_2_O_2_ levels. Although it has been shown that H_2_O_2_ exhibits better oxidative performance in more acidic environments, when using boronic acid based probes, the oxidation by H_2_O_2_ is favored in basic environments. Under basic pH, a lower amount of H_2_O_2_ is needed than in acid pH to achieve equivalent SERS readout because the reaction is favored, thus being more sensitive.

With same amount of H_2_O_2_, since the equilibrium is different for different pH values, the SERS readout will be different. Higher pH values enhance the oxidation of the H_2_O_2_ sensor thus affecting the linear ranges for H_2_O_2_ depending on the pH and resulting in different sensor sensitivity and limit of detection (LOD) (Figure 2B and 2C; SI section 2, Figure SI-10). We plotted H_2_O_2_ calibration curves for each pH value and calculated the multiplexed nanosensor’s LOD^55^ (Figure 2C). Figure SI-10 (SI section 2) shows the calibration curve functions and the LODs at different pH values for NCs@3-MPBA&4-MBA. The LOD of H_2_O_2_ at acid and neutral pH (pH 4 and pH 7) was around 10^–6^ M and close to 10^–8^ M for pH 8 and pH 9.

Summarizing the methodology, to accurately quantify cellular H_2_O_2_ dynamics with varying pH values, we first measure local pH with help of the pH calibration curve (SI section 1, Figure SI-6A). With known pH, we select the corresponding H_2_O_2_ calibration curve for this pH (Figure 2B) and obtain the local H_2_O_2_ amount. Although the multiplex sensor shows an intensity loss of around 10% of the sensitive peak 882 cm^–1^ compared with the individual nanosensor (SI section 2, Figure SI-11), the LOD results for H_2_O_2_ were in the same order of magnitude, thus being comparable.

We can conclude that our nanosensor is highly specific, being able to distinguish between molecular changes due to CT processes and specific to H_2_O_2_; however its sensitivity depends on environmental pH. The sensitivity of our nanosensor (multiplexed and individual) is pH-dependent, being maximum at high pH (7–9) and lower with decreasing pH (6–4). In general, H_2_O_2_ measurements are based on direct or indirect oxidation of a probe by H_2_O_2_;^15^ thus the pH effect on H_2_O_2_ measurements can be applied to all H_2_O_2_ sensors which are based on aromatic boronic acid coupled with fluorescence or SERS.

### Multiplex Nanosensor Cellular Internalization and Biocompatibility

**Figure 3:**
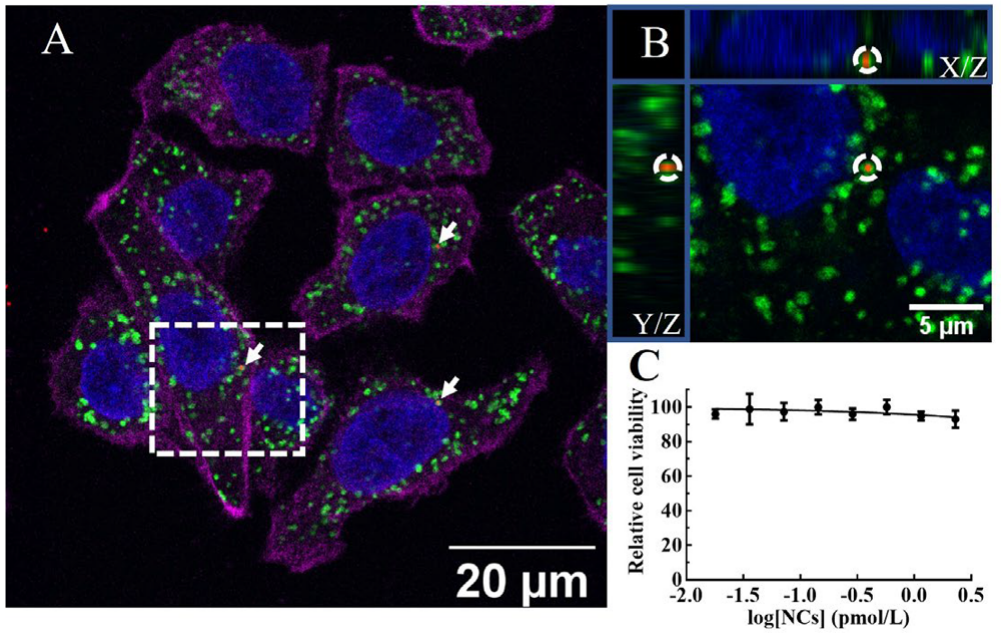
NC cellular internalization and biocompatibility. CLSM Z-scanning of the NC uptake by HT29 cells. (A) Z-scan of a cell area. Nucleus: blue; lysosomes: green; NCs: red; cytoskeleton: magenta. NCs are highlighted with white arrows. (B) Orthogonal view from different planes (*X*/*Y*; *X*/*Z*; *Y*/*Z*) of a selected area (dashed square in panel A). For the sake of clarity to confirm intralysosomal location, only the fluorescent channels of the NCs, lysosome, and nucleus are shown. (C) Cell viability is confirmed upon exposure of the cells to different NC doses.

Under steady conditions, spatial distribution of intracellular H_2_O_2_ is not equal.^13,33^ It is critical to verify the location of intracellular NCs with the purpose of analyzing the local concentration of H_2_O_2_. Depending on the nanomaterials’ physiochemical properties, different internalization pathways (phagocytosis, micropinocytosis, endocytosis) determining their location are activated.^56^ The endocytosis pathway seems to be the logical approach for our NCs (diameter ∼450 nm and SiO_2_/Au hybrid material). Figure 3 shows fluorescent labeling of different cellular organelles and the NCs. The intracellular localization of the NCs by HT29 cells after 24 h was verified to be within lysosomes (Figure 3A-3B and SI section 3, Figure SI-12A,B).

Our previous results on these NCs have demonstrated their safety profile^57^ in addition to their excellent SERS capabilities for sensing.^38,40^ In this study, we confirmed the biocompatibility of the NCs based on an unaltered mitochondrial activity. Colon cancer cells, HT29, were exposed to the NCs with a concentration range from 0.018 to 2.3 pmol/L (by number of NCs; Figure 3C and SI section 3, Figure SI-12C). As it can be observed in Figure SI-12C, the NCs were plainly visible (dark points) under an inverted optical microscope. At very high concentrations (≥1.15 pmol/L), the HT29 cells were fully covered by NCs. Under any circumstances, including [NCs] 2.3 pmol/L, no or extremely low cytotoxicity was observed after 24 h of exposure. The lethal dose killing half of the cell population (IC_50_) was 4000 pmol/L, which is significantly higher than the concentration we used in this work (0.072 pmol/L). These results confirm no effect of the NCs on the cell viability during the SERS sensing.

### Real Time and Noninvasive Multiplexing of pH and H_2_O_2_ Dynamics in Living Cells

There are data reporting the pH levels in organisms, tissues, cells, and cellular structures.^47^ However, little information exists about the cellular dynamics of H_2_O_2_ levels. To approach this issue, we tested the ability of our multiplex nanosensor to respond to real time dynamic changes of both analytes at the same time. Furthermore, we measured not only their basal levels but also induced chemical variations of the pH and H_2_O_2_ levels and confirmed the nanosensors’ dynamic response.

One of the most common and simple methods to understand intracellular H_2_O_2_ functions is to add H_2_O_2_ itself directly to the experimental system. With permeability coefficients ranging from 0.01 to 0.7 cm/min, H_2_O_2_ can permeate membrane at relatively rapid speed and establish equilibrium.^58^ To demonstrate the feasibility of mimicking intracellular cell stress upon cellular exposure to H_2_O_2_, we used a genetically encoded fluorescent probe to confirm intracellular levels (SI section 4.1, Figure SI-13A). Additionally, we observed that intracellular H_2_O_2_ reached a plateau after 10 min treatment (SI section 4.1, Figure SI-13B). We could also discard cytotoxicity issues derived from H_2_O_2_ exposure. We measured toxicity at the level of mitochondrial activity and cell membrane integrity and confirmed that exposure to H_2_O_2_ was not cytotoxic and the cells exhibited a cell viability of higher than 90% (SI section 4.1, Figure SI-13C,D). This was important to discard erratic cell stress that could affect our sensing.

Besides the cellular stimulation with H_2_O_2_, we also altered the cellular pH dynamics by treating the cells with bafilomycin A1 (SI section 4.2, Figure SI-14).^51,59,60^ bafilomycin A1 blocks the vacuolar ATPase (V-ATPase) proton pump which controls the acidification of endosomes and lysosomes. Thus, the endolysosomal vesicle pH increases upon the addition of bafilomycin A1.^61^

**Figure 4:**
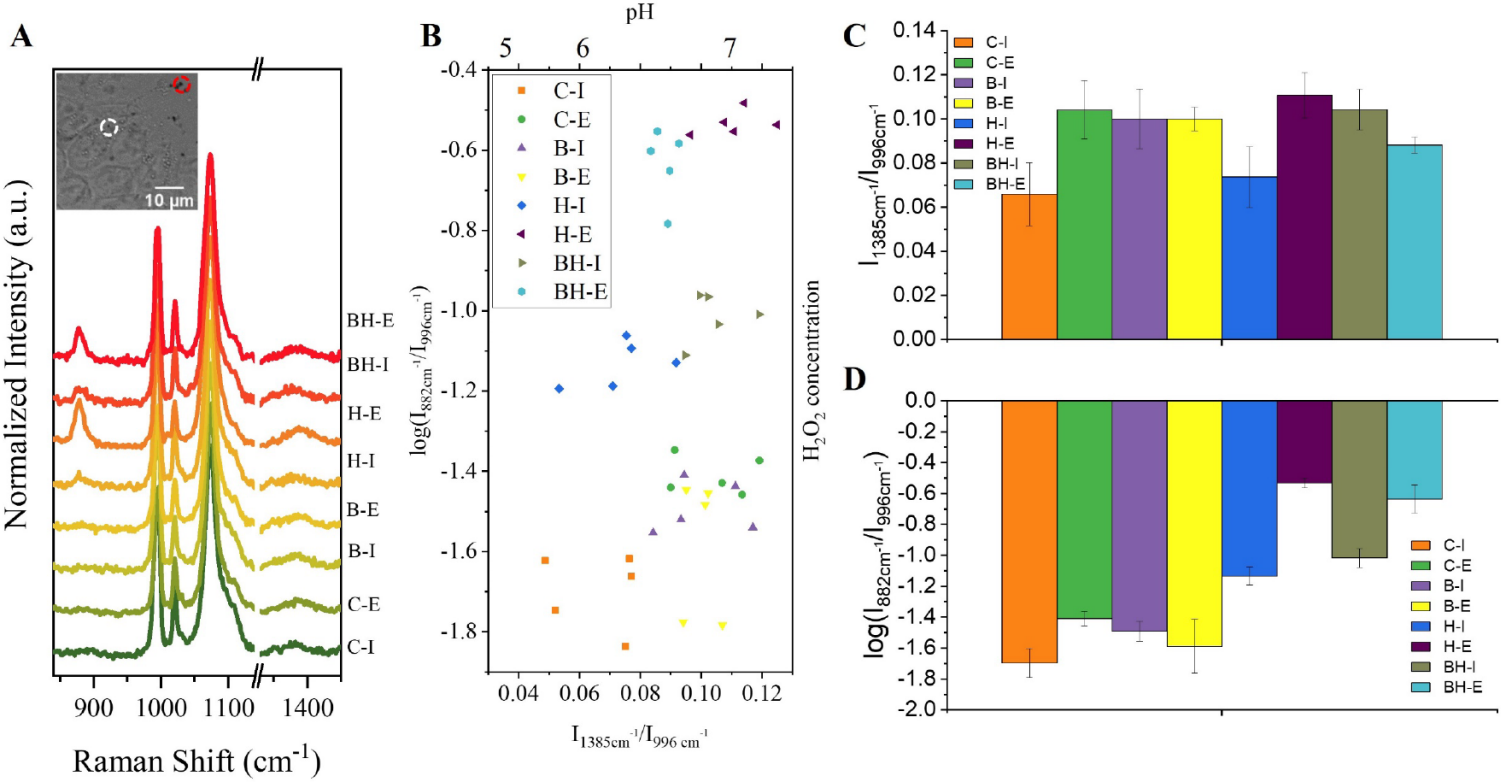
NCs@3-MPBA&4-MBA real time, live cells H_2_O_2_ and pH SERS determination. Intracellular (I) and extracellular (E) SERS spectra of untreated (control, C) and treated HT29 cell samples. The treatments were bafilomycin A1 treated (B), 10 mM H_2_O_2_ (H), and bafilomycin A1 and 10 mM H_2_O_2_ (BH). C-I: control cells’ intracellular signal; C-E: control cells’ extracellular signal; B-I: bafilomycin A1-treated cells’ intracellular signal; B-E: bafilomycin A1-treated cells’ extracellular signal; H-I: H_2_O_2_-treated cells’ intracellular signal; H-E: H_2_O_2_-treated cells’ extracellular signal; BH-I: B- and H-treated cells’ intracellular signal; BH-E: B- and H-treated cells’ extracellular signal. (A) Representative SERS spectrum for each cellular sample showing the dynamics of the H_2_O_2_- and pH-sensitive and -insensitive peaks. HT29 cell bright field images collected with the Raman microscope (inserted images). Dashed circles show internalized (white) and extracellular (red) NCs, from where the SERS signal was collected (SI section 4, Figure SI-16). (B) Intensity ratio between 1385 and 996 cm^–1^ (*I*_1385_/*I*_996_) and intensity ratio between 882 and 996 cm^–1^ (log(*I*_882_/*I*_996_)) were plotted. *I*_1385_/*I*_996_ reflects local pH values, and log(*I*_882_/*I*_996_) corresponds to local H_2_O_2_ concentrations. Each point corresponds to one capsule. Average of SERS spectra collected from 5 different probes showing pH (C) and H_2_O_2_ (D) measurements.

In this way, we have living cells with physiological and altered levels of pH and/or H_2_O_2_. We exposed these living cells to very low NCs@3-MPBA&4-MBA concentration (0.072 pmol/L) for 24 h to ensure sufficient internalization and measured intracellular and extracellular pH and H_2_O_2_ levels (Figure 4 and SI section 4.4, Figure SI-16). We fixed this experimental condition because the NCs were microscopically visible, thus it enabled us to analyze single-NC inside living cells. It also ensured whole NC illumination thus minimizing unfocused SERS signals coming from nearby NCs since the laser spot diameter (1 μm) of our Raman spectrometer was bigger than the NC diameter (approximately 400 nm). Furthermore, we observed that increasing the laser irradiation time (as is usual for biological samples) affects the stability of the molecular sensor depending on their molecular structure (SI section 4.5, Figure SI-17). High irradiation times (25 s) affected the optical response of 4-MBA (pH sensor) but not of 3-MPBA (H_2_O_2_ sensor). Possibly, high irradiation times induce long-lasting increase in the local temperature of the plasmonic nanostructure (Au) where 4-MBA is conjugated. An increased and continuous heating of 4-MBA after laser irradiation can result in a photoinduced sublimation^62^ of 4-MBA. Therefore, we can conclude that the irradiation time must be checked when selecting a Raman probe for biosensing as it may interfere with the analyte’s quantification.

Figure 4 shows the real time response of the multiplex nanosensor, NCs@3-MPBA&4-MBA, interacting with living cells. We concomitantly measured both analytes’ amount in the intracellular and extracellular milieu of different cells under physiological or altered conditions. The different cell samples were control, untreated cells exhibiting physiological levels of pH and H_2_O_2_ and cells treated with H_2_O_2_ and/or bafilomycin A to promote imbalances in H_2_O_2_ and/or pH homeostasis. Figure 4A shows the regular morphology of living cells interacting with the NCs and the SERS intensity dynamics of the sensitive and insensitive peaks of both sensors encapsulated within the NCs@3-MPBA&4-MBA (SI section 4, Figure SI-16 for the complete SERS spectrum). Figure 4B shows the distribution of pH and H_2_O_2_ values measured by each NCs@3-MPBA&4-MBA for all cell samples under different conditions, whereas Figure 4C,D shows the average value obtained from different experiments. We obtained a correlation between the ratiometric SERS response (readout) and the analyte’s amount with help of the respective calibration curves (SI section 1, Figure SI-6A, and Figure 2). We plotted in Figure 4B the values for pH because there is a direct correlation, but we could not add the H_2_O_2_ values because the amount measured depends on the environmental pH.

Let us have a look at Figure 4B and let us focus on the values obtained for the control, untreated/healthy cells (orange and green dots) exhibiting physiological levels of pH and H_2_O_2_. The extracellular NCs@3-MPBA&4-MBA (C-E sample, green dots) exhibits a ratiometric (*I*_1385_/*I*_996_) response for pH of around 0.1 (see also Figure 4C), and this value correspond to pH 7 (as indicated in the calibration curve in SI section 1, Figure SI-6A). This readout agrees with the pH of the cell’s growth media. Concomitantly, the extracellular NCs@3-MPBA&4-MBA also exhibits a ratiometric (*I*_882_/*I*_996_) response for H_2_O_2_ of around −1.4 (see also Figure 4D). Since we also know that the NCs are in an environment at pH 7, we take the H_2_O_2_ calibration curve at pH 7 (Figure 2B) and correlate the ratiometric value to its corresponding H_2_O_2_ concentration. In this case we quantified 0.8 μM of H_2_O_2_ in the extracellular milieu of control, untreated cells. Following the same methodology, the intracellular NCs@3-MPBA&4-MBA (C-I sample, orange dots) exhibits a ratiometric response for pH of 0.06–0.08 (see also Figure 4C) corresponding to local pH values ranging from approximately 5.7 to 6.5. This variation can be ascribed to the location of the NCs in different endocytic vesicles characterized by different degrees of acidification.^53^ Noteworthy that we exposed living cells to the NCs and measure individual NCs from the same and different cells in real time, thus not controlling the exact fate of the NCs inside the cells. Concomitantly, the intracellular NCs@3-MPBA&4-MBA also exhibits a ratiometric response for H_2_O_2_ of (−1.8)–(−1.6) (see also Figure 4D), which is below our limit of detection (Figure 2C and SI section 2.4, Figure SI-10). Taking the NCs@3-MPBA&4-MBA at pH 6 and its related H_2_O_2_ calibration curve, the LOD is 3.9 μM, meaning that the basal intracellular H_2_O_2_ concentration of healthy cells is below this value.

Let us continue in Figure 4B and check the results obtained for the “unhealthy”/treated cells where analyte imbalances have been chemically induced. Let us focus first on the cells with an altered pH homeostasis exhibiting alkalinization of the endolysosomal compartments and analyze the pH and H_2_O_2_ amounts measured by the intracellular (B-I) and extracellular (B-E) NCs@3-MPBA&4-MBA (yellow and light purple triangles). Compared to the control, healthy cells, the extracellular pH values were unaltered (pH 7); however the pH of the endolysosomal vesicles transporting the NCs@3-MPBA&4-MBA was higher (6.5–7.5). These results agree with the alkalinization process we induced in the cells. We could not establish differences in the extracellular and intracellular H_2_O_2_ concentrations because the values obtained are below our NCs@3-MPBA&4-MBA LOD (0.77 μM at pH 7) (Figure 2C and SI section 2.4, Figure SI-10).

The workflow of the measurement and the values obtained for all cellular samples is presented in Table 1 (SI section 4, Table SI-2).

**Table 1:** NCs@3-MPBA&4-MBA Multiplexing Workflow^a^

				unhealthy cells					
		healthy cells		endolysosomal alkalinizatlon		oxidative stress		oxidative stress and endolysosomal alkalinization	
		extracellular (C-E)	intracellular (C-I)	extracellular (B-E)	intracellular (B-I)	extracellular (H-E)	intracellular (H-I)	extracellular (BH-E)	intracellular (BH-I)
pH	SERS intensity ratio (*I*_1385_/*I*_996_)	0.1	0.05–0.08	0.1	0.08–0.12	0.10–0.12	0.05–0.09	0.09	0.09–0.12
	pH value shown in the calibration curve	7	5.7–6.5	7	6.5–7.5	7–7.5	5.7–6.7	∼7	6.7–7.5
H_2_O_2_	SERS intensity ratio (log(*I*_882_/*I*_996_))	–1.4	∼(−1.7)	(−1.8)–(−1.5)	∼(−1.5)	∼(−0.5)	(−1.1)–(−1.2)	–0.6	–1.0
	log[H_2_O_2_] (M) in the calibration curve (pH calibration curve used)	–6.1	<(−6) (pH 6)	<(−7.6)–(−6.5)	–6.5 (pH 7)	–2.5	(−3.9)–(−4.3) (pH 6)	–3	–4.5 (pH 7)
	[H_2_O_2_]	0.8 μM	<3.9 μM (LOD)	<0.77 μM (LOD)	<0.77 μM (LOD)	3.2 mM	126–50 μM	1 mM	32 μM

a Ratiometric SERS measurements providing pH and H_2_O_2_ quantitative values of healthy (physiological levels) and unhealthy cells showing an altered pH homeostasis (cellular alkalinization), H_2_O_2_ homeostasis (oxidative stress), and both conditions.

Let us analyze now the “unhealthy”/treated cells exhibiting oxidative stress. The extracellular (Figure 4B–D; H-E, dark purple dots) and intracellular (H-I, dark blue dots) pH values measured by the NCs@3-MPBA&4-MBA were very similar to the healthy cells. Thus, we can conclude that oxidative stress had little effect on the local pH. Concerning H_2_O_2_, the intracellular H_2_O_2_ levels were higher (50–126 μM) than the control cells (<3.9 μM) and around 30 times lower than extracellular concentration (Table 1 and table SI-2). Interestingly, we measured a lower extracellular H_2_O_2_ amount (3.2 mM) than the amount added (10 mM) (Table 1 and Table SI-2). This could be explained by an activation of the cellular metabolism to rapidly remove extracellular H_2_O_2_.^36^

Finally, we multiplex extracellular and intracellular pH and H_2_O_2_ dynamics in “unhealthy” cells exhibiting oxidative stress and cellular alkalinization (Figure 4B; BH-E and BH-I, light blue and olive-green dots, respectively). Consistent with the results obtained before and the literature, the extracellular and intracellular pH values were pH 7 and alkalinized pH 6.7–7.5, respectively (Table 1). Comparing the H_2_O_2_ values in cells showing oxidative stress and alkalinization (BH-E and BH-I) and only oxidative stress (H-E and H-I), the levels were different but always in the same order of magnitude. For example, the extracellular H_2_O_2_ (BH-E) levels were 3 times lower but still in the mM range (H-E) whereas the intracellular levels (BH-I) were smaller but still in the μM range (H-I) (Table 1). They lowered from 126–50 μM to 32 μM (Table 1). Most probably, we have induced an effect on the cellular redox metabolism that may be sensitized by pH imbalances. As expected, intracellular (BH-I) H_2_O_2_ levels were significantly higher than under physiological conditions (C-I).

These results conclude that both homeostasis mechanisms are well preserved and do not show cross talk. Oxidative stress has no effect on pH homeostasis and cellular alkalinization has no effect on ROS species H_2_O_2_ homeostasis.

We cannot guarantee that the values are accurate and exact because we have no reference to compare and there might be some cellular or nanomaterials phenomena that we have not considered. Therefore, these results although they are quantitative, should be taken as relative and not absolute. They demonstrate a tendency in the analyte’s dynamics between cellular spaces and cell conditions.

## Conclusions

We have synthesized a complex nanocapsule composed of plasmonic gold nanoparticles functionalized with Raman probes placed on the inner surface of the silica shell. The NCs exhibit interparticle hot-spots in their internal surface and silica shell preventing physicochemical interaction between the gold nanoparticles and macromolecules from biological media interfering with the SERS signal. We selected 3-MPBA as H_2_O_2_ reporter and 4-MBA as pH reporter and validated the performance for H_2_O_2_ and pH quantification. To obtain equal signal intensities, we established a 15:1 ratio between 3-MPBA and 4-MBA. The signal is ratiometric (analyte-sensitive vs. -insensitive band), to avoid inconsistencies from external parameters like batch-to-batch differences, different amount of internalized nanocapsules or changes in the plasmonic interface over time. 4-MBA is thermolabile, thus the irradiation of the nanocapsules must be kept below 25 s to preserve the response.

The 3-MPBA H_2_O_2_ readout depends on the local pH. The complexation of boronic acid with three hydroxyl groups in an alkaline pH environment enhanced the B–C bond cleavage sensitivity, which favors 3-MPBA oxidation by H_2_O_2_. Therefore, we can conclude that the sensitivity of H_2_O_2_ sensors based on aromatic boronic acid is pH dependent. In our case, the reporter’s signal (multiplexed and individual) is maximum at high pH (7–9) and lower at acidic pH (6–4). We could confirm that this effect is not promoted by the coexistence of both reporters on the same plasmonic surface nor by a pH-mediated cross talk of the H_2_O_2_ signaling bands. We can also conclude that our nanosensor is highly specific, being able to distinguish between molecular changes due to charge transfer processes, and specific to H_2_O_2_. We validated our multiplex nanosensor’s performance by concomitantly quantifying both analytes (first pH and based on the value then H_2_O_2_) under physiological and pathological (oxidative stress and/or cellular alkalinization) conditions. NCs@3-MPBA&4-MBA were able to quantify pH and H_2_O_2_ dynamics extra- and intracellularly.

We have a set of SERS probes for NO, pH, and ROS (H_2_O_2_) based on the same nanomaterial system and readout (SERS); however they behave molecularly differently. Each reporter exhibits different molecular properties affecting their readout. For example, 3-MPBA has a lower Raman cross section than 4-MBA and its sensitivity depends on environmental pH whereas 4-MBA is more thermolabile than 3-MPBA. We can conclude how important it is to rationally design your nanomaterial based on the application requirements and considering the physicochemical properties of each of the building blocks forming the nanomaterial.

## Supporting Information Available

More detailed information, raw data, and complementary results on nanosensor synthesis and characterization, influence of physiological pH levels on sensitivity, cellular internalization and biocompatibility, and multiplexing of pH and H_2_O_2_ dynamics in living cells (PDF)
