# Supplementary material for: Real Time and Spatiotemporal Quantification of pH and H2O2 Imbalances with a Multiplex Surface-Enhanced Raman Spectroscopy Nanosensor
Source: ACS Mater Au. 2023 Feb 15;3(2):164–75. doi: 10.1021/acsmaterialsau.2c00069 (PMC9999477; doi:10.1021/acsmaterialsau.2c00069)
Supplement: Supplementary file 1 — mg2c00069_si_001.pdf [file mg2c00069_si_001.pdf]

Real time and spatiotemporal quantification of pH and H_2O_2 imbalances with a multiplex SERS nanosensor

Can Xiao[1], Victor Izquierdo-Roca[2] and Pilar Rivera-Gil[1] *

[1] Dr. C. Xiao, and Dr. P. Rivera-Gil
Department of Experimental and Health Sciences
University Pompeu Fabra
Carrer Doctor Aiguader 88, 08003 Barcelona, Spain
E-mail: pilar.rivera@upf.edu

[2] Dr. V. Izquierdo-Roca
Catalonia Institute for Energy Research (IREC)
Jardins de les Dones de Negre 1, 08930 Sant Adrià del Besòs, Barcelona, Spain

SUPPORTING INFORMATION

Contents

Section 1: Multiplex nanosensor's synthesis and characterization. **2**

1.1 Synthesis of the nanosensors based on silica coated plasmonic LbL (AuNPs@NCs). 2

1.2 Validating the nanosensor for the ROS specie, H_2O_2 (NCs@3-MPBA). 3

1.3 Validating the nanosensor for pH sensing (NCs@4-MBA).......... 5

1.4 Synthesis and characterization of the multiplex nanosensor (NCs@3-MPBA&4-MBA).......... 6

Section 2: Influence of physiological pH levels on boronate-based H_2O_2 sensors' sensitivity. **9**

2.1 NCs@3-MPBA's response to H_2O_2 dynamics depends on environmental pH values. 9

2.2 SERS response of NCs@3-MP (the oxidized form of 3-MPBA) does not depend on environmental pH. 9

2.3 The pKa of 3-MPBA determines the sensitivity of boronic acid-based H_2O_2 sensors. 10

2.4 NCs@3-MPBA&4-MBA's LOD depends on pH. 11

Section 3: Multiplex nanosensor's cellular internalization and biocompatibility. **13**

Section 4: Real time and non-invasive multiplexing of pH and H_2O_2 dynamics in living cells. **14**

4.1 Chemical alteration of the cellular H_2O_2 homeostasis to induce oxidative stress. 14

4.2 Inducing intracellular alkalinization to alter pH homeostasis. 14

4.3 Cellular H_2O_2 sensing with NCs@3-MPBA. 15

4.4 Cellular multiplexing of H_2O_2 and pH imbalances with NCs@3-MPBA&4-MBA. 16

4.5 High irradiation time causes photosublimation of 4-MBA which results in an altered spectrum. 17

References **19**

Section 1: Multiplex nanosensor's synthesis and characterization.

1.1 Synthesis of the nanosensors based on silica coated plasmonic LbL (AuNPs@NCs).

Figure SI-1 shows the functional nanosensors' characterization during different steps of the synthesis with Transmission Electron Microscopy (TEM) (A-C), Dynamic Light Scattering (DLS) (D-E), and SERS (F). Briefly, the synthesis of the NCs is performed wrapping polyelectrolytes of opposite charge (PSS and PAH) layer by layer (LbL) onto a template (polystyrene, PS) being the last layer, a deposition of gold seeds. We grow a final silica (SiO_2) shell to protect the plasmonic nanostructure and to preserve the SERS signal. Afterwards, we dissolve the PS template chemically and grow the gold seeds inside into a plasmonic nanostructure showing a characteristic LSPR. DLS measurements show a hydrodynamic size of 467.6 nm and PDI 0.044 (SI-1D) and a ζ-potential mean value -36.7 mV.

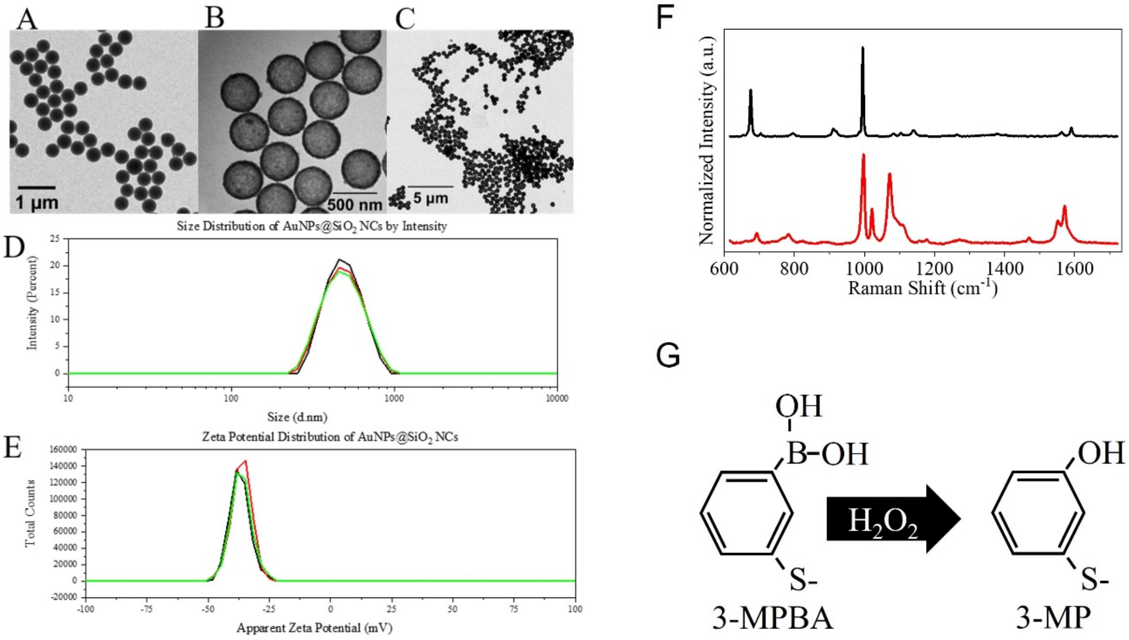

Figure SI-1: Nanocapsules (NCs) characterization. **(A)** TEM image of the template, PS beads, coated with the LbL. **(B)** TEM image of hollow NCs after template dissolution (Au seeds@SiO_2 NCs). **(C)** TEM image of the nanosensor *i.e.*, the NCs after growing the Au seeds into Au nanoparticles (NPs) exhibiting hot spots required for SERS. **(D)** NCs size measurements (3 repetitions). **(E)** NCs' ζ-potential measurements (3 repetitions). **(F)** Raman spectrum of 3-MPBA in powder (black line) and SERS spectrum of 3-MPBA conjugated to the AuNPs@SiO_2 NCs (red line). **(G)** Scheme of the oxidation process by H_2O_2 of 3-MPBA to 3-MP.

The hollow plasmonic NCs are functionalized with the Raman probes for sensing. We selected 3-MPBA as a H_2O_2 sensor molecule because it can be oxidized into 3-hydroxyl thiophenol (3-MP) in the presence of H_2O_2 showing new SERS characteristic bands of 3-MP[1] (figure SI-1F). Figure SI-1G shows the Raman spectrum of 3-MPBA in powder form (black line) and SERS spectrum of 3-MPBA modified NCs (NCs@3-MPBA; red line). The spectral fingerprint differences between both spectra are the consequence of the surface selection rules and the surface enhancement due to the media interaction and the resonance coupling occurring when 3-MPBA is adsorbed onto the NCs' metallic surface. In this context, the disappearance of the peak at 910 cm^{-1} associated to the vibration mode of the thiol group (-SH) is a confirmation of the

deprotonation of this group and a consequence of 3-MPBA bonding to the NCs[2]. Moreover, an intense SERS spectrum of 3-MPBA confirming the successful functionalization of the NCs appears[1]. Characteristic features include the bands at 783 cm^{-1} assigned to C-H out of plane bending mode, at 996 cm^{-1} resulting from C-C in plane bending mode, at 1020 cm^{-1} attributed to C-H in plane bending mode, at 1070 cm^{-1} issued from C-C in plane bending coupled with C-S stretching modes, at 1553 cm^{-1} referred to non-totally symmetric benzene ring stretching mode, and at 1570 cm^{-1} imputed to totally symmetric benzene ring stretching mode.

Table SI-1 shows a comparison of other systems different than 3-MPBA published for hydrogen peroxide detection. It includes commercial boronate based fluorescent probes, genetically encoded fluorescent probes, and other SERS system. In generally, SERS assays show better sensitivity than fluorescence assays, and great potential in multiplex analysis because of the easy functionalization of SERS probes.

Sensors	Sensing principle	Sensitivity	Features	Reference
Peroxy Orange 1	Boronate based fluorescence assay	N/A	H_2O_2 detection under oxidative stress conditions.	Ref. [3]
ROS-Glo™	Boronate based luminescent assay	N/A	Enhanced sensitivity than boronate based fluorescent probes.<br>Limited to the extracellular compartment.<br>Reacts with $ONOO^-$.	Ref. [4,5]
Hyper-Probe	Genetically encoded probe for fluorescence assay	250 nM	Subcellular resolution for H_2O_2 detection.<br>pH interferences.	Ref. [6]
roGFP-Orp1	Genetically encoded probe for fluorescence assay	750 nM	Subcellular resolution for H_2O_2 detection.<br>Cellular reducing capacity interference.	Ref. [5,7]
phenylboronic acid pinacol ester	Boronate based SERS assay	200 nM	Mitochondrial H_2O_2 detection.	Ref. [8]
3-Mercaptophenyl boronic acid	Boronate based SERS assay	45nM - 48µM with pH from 9 to 4	Simultaneous detection of pH and H_2O_2.	ours

Table SI-1: Summary of systems for hydrogen peroxide detection.

1.2 Validating the nanosensor for the ROS specie, H_2O_2 (NCs@3-MPBA).

To confirm the H_2O_2 sensing capability of NCs@3-MPBA, we first elucidated the peaks sensitive to H_2O_2 (figure SI-2). To emphasize the spectra modification, a statistical analysis of the spectra variation based on

standard deviation was conducted and shown in figure SI-2A (blue spectrum), allowing us to detect significant spectra variation. With variation higher than 25 %, they are sensible to H_2O_2 concentration.

A

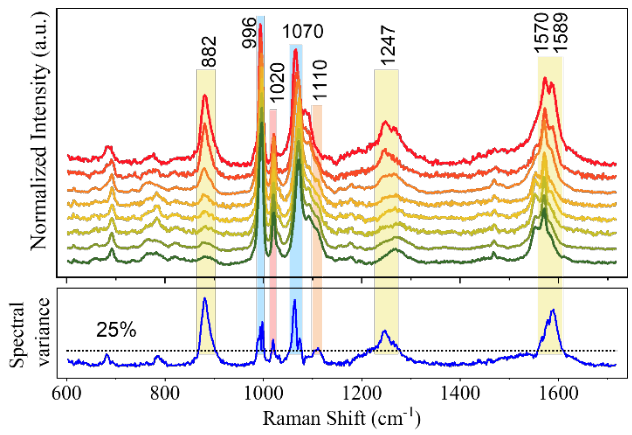

B

Without H_2O_2		*With H_2O_2*		
**3-MPBA Assignation**	**Raman shift**	**Raman shift**	**Intensity evolution**	**3-MP & 3-MPBA Assignation**
	-	882	Increasing	benzene ring stretching
CC in-plane bending	996	994	equal	CC in-plane bending
CH in-plane bending	1020	1020	Decreasing	
CH in-plane bending + CS stretching	1070	1063	Equal	CH in-plane bending + CS stretching
CH in-plane bending	1110	1110	Decreasing	
		1247	Increasing	CH in-plane + CH bending
non-totally symmetric benzene ring stretching	1553	1553	Decreasing	non-totally symmetric benzene ring stretching
totally symmetric benzene ring stretching	1570	1570	equal	totally symmetric benzene ring stretching
	-	1589	Increasing	totally symmetric benzene ring stretching

Figure SI-2: SERS response characterization of the H_2O_2 nanosensors, NCs@3-MPBA. (A) SERS spectra characterization and spectral variance of NCs@3-MPBA dispersed in pH7 phosphate buffer with different H_2O_2 concentrations (10^{-2} M, 10^{-3} M, 10^{-4} M, 10^{-5} M, 10^{-6} M, 10^{-7} M, 10^{-8} M, and 0 M) (from red to green). Each spectrum was the mathematical average of 5 spectra obtained from 5 different NCs@3-MPBA. **(B)** Table showing the assignment of the NCs@3-MPBA main SERS peaks and their evolution in the presence and absence of H_2O_2.

These spectra variations can be categorized into three different types: i) peak intensity reduction associated to bond relative concentration reduction (peaks at 1020 and 1110 cm^{-1}, orange shadow), ii) peak intensity increase attributed to bond relative H_2O_2 concentration increase (peaks at 882 and 1240 cm^{-1} and broad band at 1550-1600, yellow shadow areas), and iii) peaks shift associated to modification in the bond close environment (peaks at 996 and 1070 cm^{-1}). The emerged peaks at 882 and 1589 cm^{-1} with the contribution of H_2O_2 concentration are assigned to the benzene ring stretching (ν_{12}) and the totally symmetric ring stretching (ν_{89}) of 3-MP, respectively. Figure SI-2B included a summary of the assignment of the peaks in the SERS spectra and the evolution of 3-MPBA before and after the oxidation by H_2O_2. If the H_2O_2 concentration increases, the SERS intensity of these bands also increases because of the oxidation of 3-MPBA to 3-MP and the conversion of boronate to hydroxyl functional group (figure SI-1G), in consistency with previous studies[1]. Moreover, the oxidation of 3-MPBA did not change significantly the molecular orientation of the mercaptobenzene group on the gold surface since we did not monitor a large set of different perturbations on the SERS spectra of the mercaptobenzoyl moiety[9] (figure SI-1F). We did observe that the intensity of the peak associated with the C-C in plane bending mode at 996 cm^{-1} was not affected by the oxidation of the molecule. This invariance of the intensity allows us to use this contribution as a reference band for ratiometric analysis. This independence is the consequence that C-C bonds are not created or destroyed during the oxidation process. There is a minimal blue shift (2 cm^{-1}) of the C-C peak associated to the modification of the environment of the C-C bond. However, this effect does not compromise to use of the peak intensity as internal calibration to correct signal fluctuation and to minimize the impact of external parameters such as NCs batch-to-batch variability or different cellular loading.

Then we confirmed a dynamic responsiveness by analyzing the reaction time of NCs@3-MPBA interacting with H_2O_2 in saline buffer (pH 7) (figure SI-3A). We set up the timing for future experiments at 30 min. Then, we measured the SERS spectra of the NCs@3-MPBA dispersed in cells growth medium (pH 7.2-7.4, figure SI-3B), containing variable amounts of H_2O_2. Figure SI-3C shows the relations between intensity ratio of 882 cm^{-1} and 996 cm^{-1} (log [I_{882}/I_{996}]) and the concentration of H_2O_2 (log[H_2O_2]) of the NCs@3-MPBA dispersed in phosphate buffered saline (black line) and in cell growth media (red line). Both calibration curves were similar, proving the value of our sensor in biological environments.

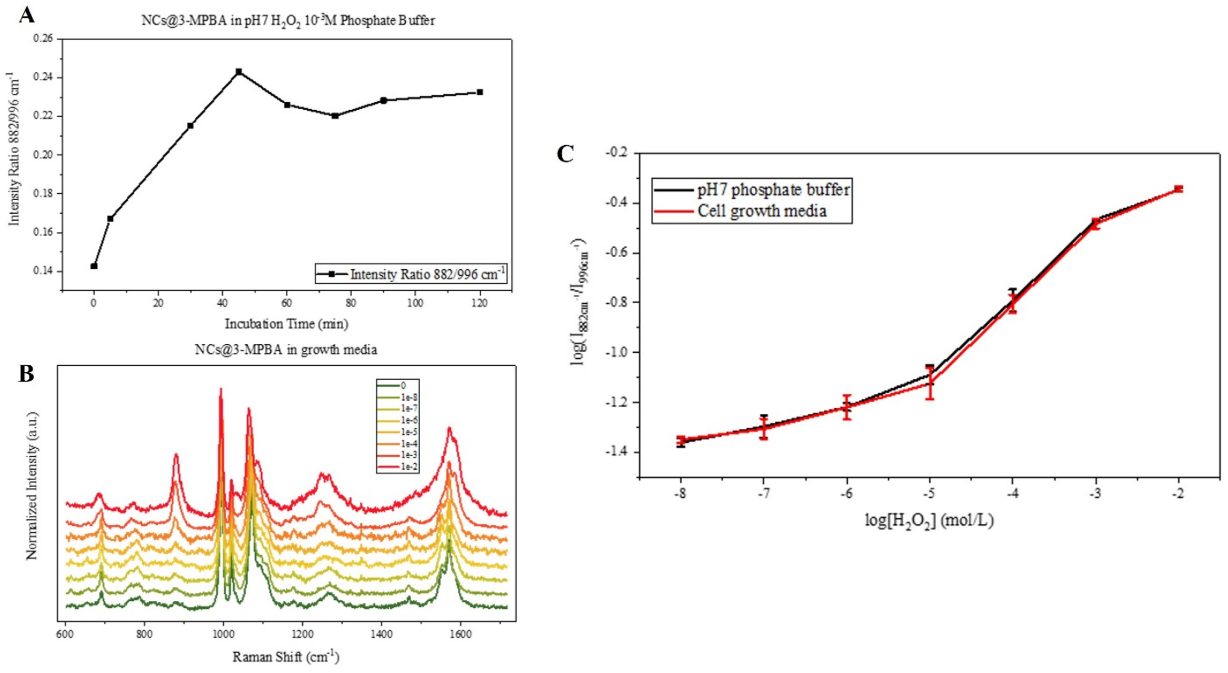

Figure SI-3: SERS response characterization of the H_2O_2 nanosensors, NCs@3-MPBA, in different biological media. (A) Kinetic study based on the intensity ratio of 882 cm^{-1} to 996 cm^{-1} by measuring NCs solution mixed with 10^{-3} M H_2O_2 in pH7 phosphate buffered saline. **(B)** NCs@3-MPBA dispersed in cells growth media with different H_2O_2 concentrations (10^{-2} M, 10^{-3} M, 10^{-4} M, 10^{-5} M, 10^{-6} M, 10^{-7} M, 10^{-8} M) and without H_2O_2 (from red to green). Each spectrum was the average of 5 spectra gotten from 5 different NCs@3-MPBA. **(C)** Calibration curves of NCs@3-MPBA determining H_2O_2 in phosphate buffer and in cells growth media followed the same trend, indicating that calibration curves gotten in buffer system can be easily used in growth media environment.

1.3 Validating the nanosensor for pH sensing (NCs@4-MBA).

4-MBA has been previously used for pH sensing with SERS[10,11] since the ratiometric intensity signal of COO^- stretching vibration mode can be calibrated for pH sensing. The peaks at around 1075 cm^{-1} and 1590 cm^{-1} correspond to aromatic ring vibrations. The peaks at around 1385 cm^{-1} and 1700 cm^{-1} are attributed to symmetric carboxyl stretching mode and C=O stretching vibrations of non-dissociated COOH groups, respectivel[10,12].
Following previously results from us and other groups, we conjugate the NCs with 4-MBA for pH sensing and we further proofed that the pH signaling was not altered by the presence of H_2O_2. As shown in figure SI-4, we collected SERS spectra of 4-MBA modified NCs (NCs@4-MBA) in phosphate buffered saline under pH 4 and pH 7 with and without H_2O_2. We did not observed changes in the vibrational modes, confirming that the commonly used pH sensitive peak at around 1385 cm^{-1} can be used for pH sensing even in the presence of H_2O_2.

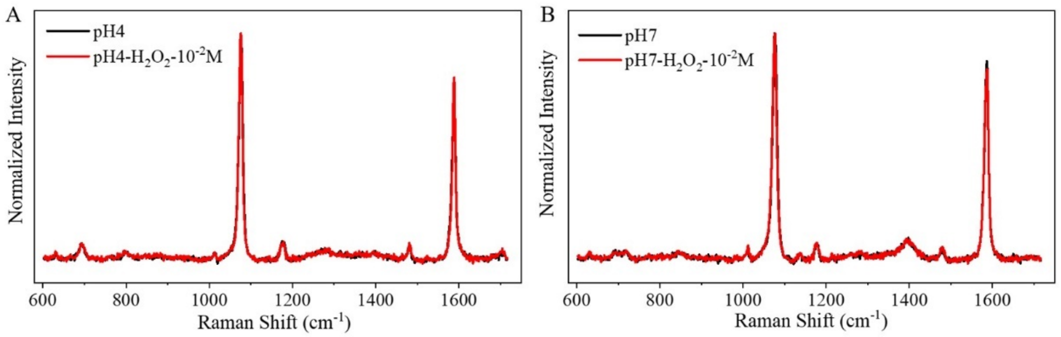

Figure SI-4: SERS spectra of NCs@4-MBA dispersed in buffer with and without H_2O_2. (A) SERS spectra of NCs@4-MBA in pH 4 buffer without H_2O_2 (black line) and with 10^{-2} M H_2O_2 (red line, incubated after 2 hours); (B) SERS spectra of NCs@4-MBA in pH 7 buffer without H_2O_2 (black line) and with 10^{-2} M H_2O_2 (red line, incubated after 2 hours).

1.4 Synthesis and characterization of the multiplex nanosensor (NCs@3-MPBA&4-MBA).

3-MPBA and 4-MBA modified NCs (NCs@3-MPBA&4-MBA) were synthesized following the same procedure as described before. The general approach was to incubate the synthesized NCs with diluted feedstock solution of the two Raman probes (3-MPBA and 4-MBA) in ethanolic solution. The surface composition of functionalized NCs was monitored by SERS. By simply comparing the strong bands intensities at 996 cm^{-1} and 1075 cm^{-1}, the relative proportions on NCs surface of each probe can be estimated. The peak around 996 cm^{-1} is attributed to C-C in plane bending mode of 3-MPBA, and the peak around 1075 cm^{-1} attributed to aromatic ring vibrations of 3-MPBA and 4-MBA. We merged the SERS spectra of 3-MPBA and 4-MBA with the ratio 1:1 and calculated the intensity ratio between 1075 cm^{-1} to 996 cm^{-1} (I_{1075}/I_{996}) as shown in Figure SI-5A. When I_{1075}/I_{996} is around 1.55, the signal proportion of both Raman probes will be equivalent. The surface composition is the result of competition for surface site between 4-MBA and 3-MPBA. Thus, for the synthesis of the multiplex sensor, we adjust the concentration ratio between 4-MBA and 3-MPBA to achieve equivalent signals. Figure SI-5B shows the SERS spectra of a series of NCs prepared with different 3-MPBA and 4-MBA ratios, and the zoomed are of the spectra showing the variations of the selected bands. The relative intensities of the marker bands at 1075 cm^{-1} to 996 cm^{-1} changed dramatically. Only 4-MBA bands at 1075 cm^{-1} appeared in the spectra at lower concentration ratio. Figure SI-5C shows the relation of I_{1075}/I_{996} against the ratios of the two Raman probes. When the 3-MPBA:4-MBA ratio increased from 5 to 20 times, I_{1075}/I_{996} decreased from 3 to 1.4. We selected a ratio of 15:1 (3-MPBA:4-MBA) as working condition, because the I_{1075}/I_{996} was round 1.55, thus indicating equivalent signals for both probes.

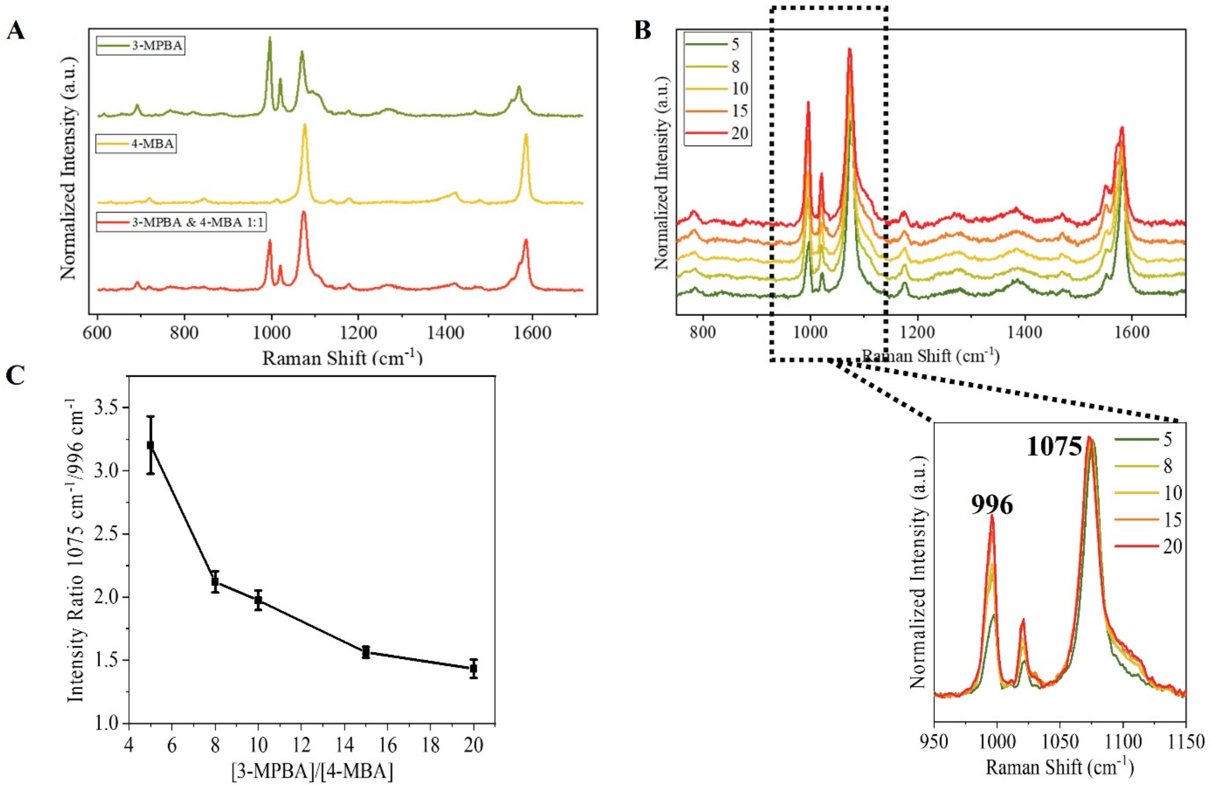

Figure SI-5: Calculating the respective amounts of 3-MPBA and 4-MBA for the conjugation of the NCs to achieve equal signal intensities. **(A)** 3-MPBA and 4-MBA spectra are merged mathematically one by one to mimic an equivalent amount of both probes attached to the NCs. **(B)** SERS spectra of NCs@3-MPBA&4-MBA with different 3-MPBA and 4-MBA concentration ratios ([3-MPBA] / [4-MBA] =5; 8; 10; 15; 20 / 1). Each plotted spectrum is the average of 5 spectra obtained from 5 different NCs@3-MPBA&4-MBA. The zoomed SERS spectra shows the bands of interest at 996 cm^{-1} and 1075 cm^{-1}. **(C)** Intensity ratios between 1075 cm^{-1} and 996 cm^{-1} as a function for 3-MPBA and 4-MBA modification. Error bars represent the standard deviations of five measurements.

We then measured the response of NCs@3-MPBA&4-MBA to different concentration of H_2O_2 and pH to confirm the preservation of the SERS signal for both probes (figure SI-6). The signal changes in NCs@3-MPBA&4-MBA agree with the phenomenon we observed with the NCs@3-MPBA *i.e.*, a $\log(I_{882}/I_{996})$ increase with both H_2O_2 and pH (figure SI-2-SI-3). Bands at around 996 and 1075 cm^{-1} are attributed to aromatic ring vibrations, which are not sensitive to pH neither H_2O_2. Bands at around 1385 cm^{-1} and 1700 cm^{-1} are related to carboxyl group vibrations and are only sensitive to pH (figure SI-4). Intensity at 1385 cm^{-1} increased along with pH increase, while intensity at 1700 cm^{-1} decreased when pH increased. We chose the intensity ratio between the well-known sensitive peak at around 1385 cm^{-1} [10] and the insensitive peak at 996 cm^{-1} (I_{1385}/I_{996}) to obtain the calibration curves for pH measurement (figure SI-6A). NCs@3-MPBA&4-MBA were sensitive to changes in the pH ranging from 5 to 7, presenting typical Henderson–Hasselbalch plots[13], which is in agreement with the SERS results published of 4-MBA covered gold nanoparticles[10]. The NCs@3-MPBA&4-MBA maintain the sensitivity of the band at 882 cm^{-1}, corresponding to the benzene ring stretching mode of 3-MPBA, to H_2O_2. Its intensity increases along with H_2O_2 (figure SI-6B).

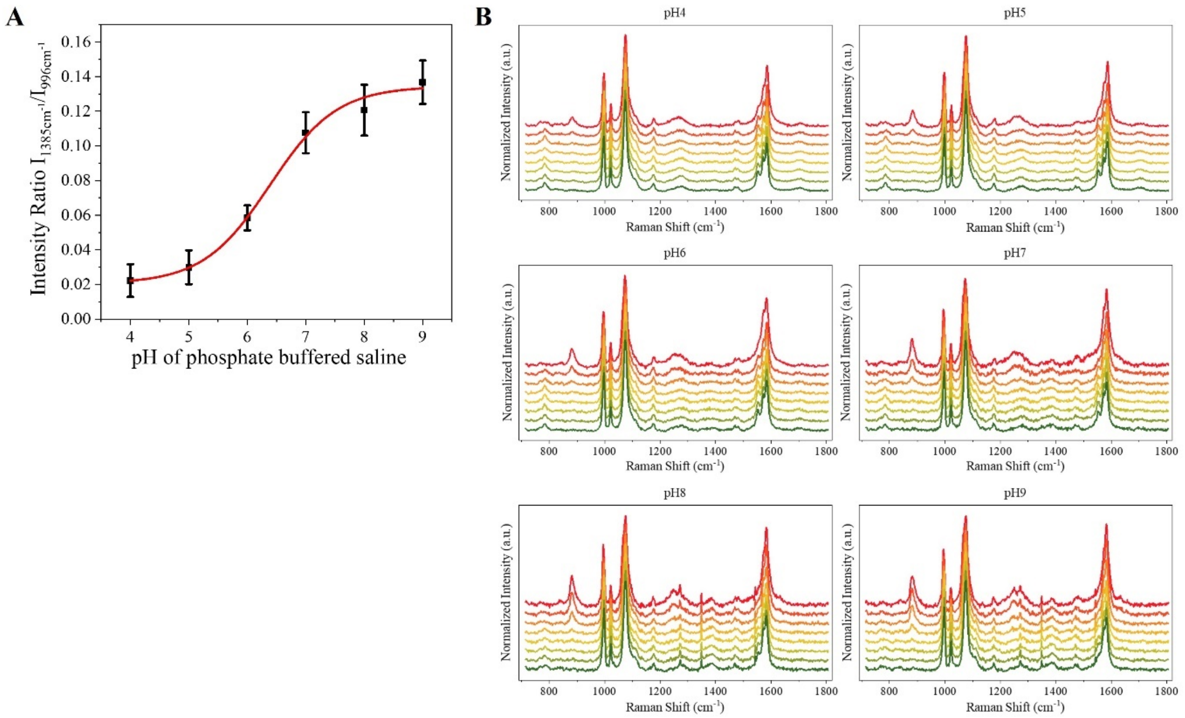

Figure SI-6. NCs@3-MPBA&4-MBA's responsiveness to H_2O_2 and pH dynamics. (A) Calibration curves of NCs@3-MPBA&4-MBA for pH in phosphate buffer (from 4 to 9). The averages and standard deviations were calculated with the spectra in the presence and absence of H_2O_2 within the same pH. **(B)** SERS spectra of NCs@3-MPBA&4-MBA in phosphate buffer at different pH and H_2O_2 concentrations, with pH ranging from 4 to 9, and H_2O_2 from 10^{-2} M to 10^{-8} M and without H_2O_2 (from red to green, H_2O_2 concentration 10^{-2} M, 10^{-3} M, 10^{-4} M, 10^{-5} M, 10^{-6} M, 10^{-7} M, 10^{-8} M, and without H_2O_2), showing how the intensity at 882 cm^{-1} decreased from red to green and the intensity at 1385 cm^{-1} varied along with pH. Each spectrum was the average of 5 spectra gotten from 5 different NCs@3-MPBA&4-MBA.

Section 2: Influence of physiological pH levels on boronate-based H_2O_2 sensors' sensitivity.

2.1 NCs@3-MPBA's response to H_2O_2 dynamics depends on environmental pH values.

Figure SI-7 shows how the SERS intensity ratios $\log(I_{882}/I_{996})$ of NCs@3-MPBA varied at different pH and H_2O_2 concentrations, ranging from pH 4 to pH 9 and H_2O_2 concentrations from 10^{-2} M to 10^{-8} M. The SERS intensity ratio $\log(I_{882}/I_{996})$ increases along with increasing H_2O_2 concentrations. Moreover, $\log(I_{882}/I_{996})$ also increased when the pH became more alkaline under same H_2O_2 concentration. For example, I_{882}/I_{996} at pH 9 and $[H_2O_2] = 10^{-6}$ M was approx. 4 times higher than at pH 4. These results indicate an influence of pH on H_2O_2 quantification although exclude cross talks since the H_2O_2 sensitive bands are not affected in the absence of the analyte.

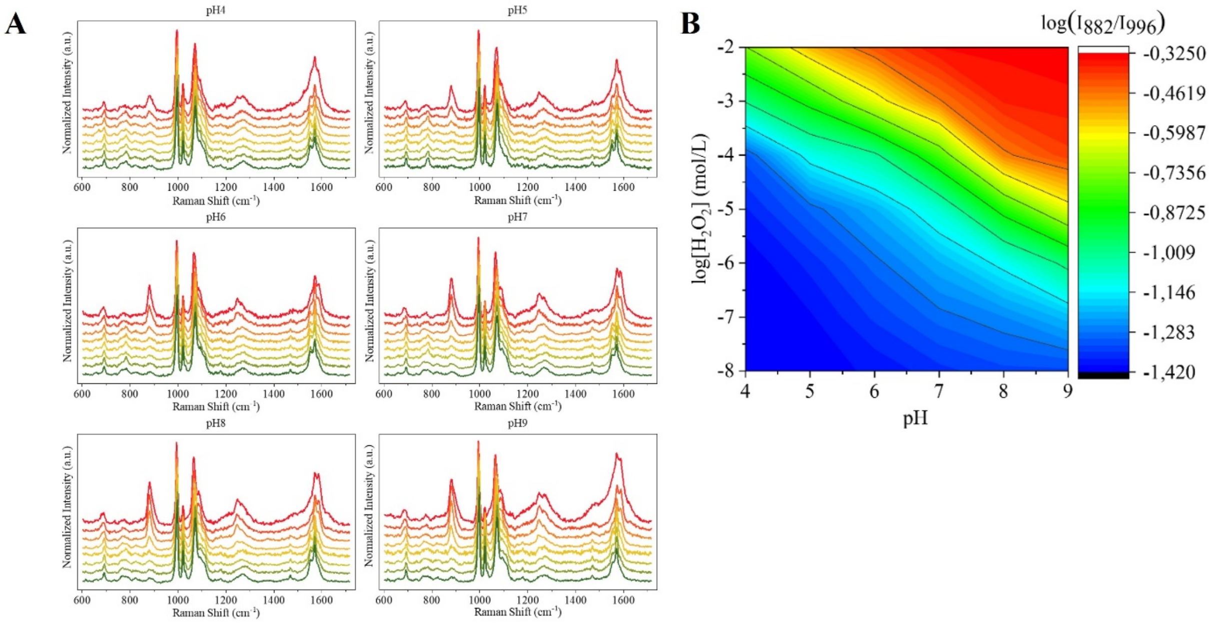

Figure SI-7: NCs@3-MPBA's response to H_2O_2 and pH. (A) SERS spectra in phosphate buffer at different pH and H_2O_2 concentrations, with pH ranging from 4 to 9, and H_2O_2 from 10^{-2} M to 10^{-8} M and without H_2O_2 (from red to green H_2O_2 concentration 10^{-2} M, 10^{-3} M, 10^{-4} M, 10^{-5} M, 10^{-6} M, 10^{-7} M, 10^{-8} M, 0 M), showing how the intensity at 882 cm^{-1} decreased from red to green, respectively. Each spectrum was the average of 5 spectra obtained from 5 different NCs@3-MPBA. **(B)** The 3D matrix shows how $\log(I_{882}/I_{996})$ changes with H_2O_2 under different pH. Values were calculated based on SERS spectra of NCs@3-MPBA dispersed in phosphate buffer with pH ranging from 4 to 9, and H_2O_2 from 10^{-2} M to 10^{-8} M. Each point is the average of five probes.

2.2 SERS response of NCs@3-MP (the oxidized form of 3-MPBA) does not depend on environmental pH.

H_2O_2 oxidized 3-MPBA into 3-MP. We functionalized AuNPs@NCs with 3-MP and measured the SERS spectra at different pH values in the absence of H_2O_2 to check the stability and specificity of the signal (figure SI-8). We observed no change (*e.g.,* band shift, intensity ratios, among others) in its vibrational mode, thus concluding no cross talk and confirming signal specificity of 3-MPBA to H_2O_2.

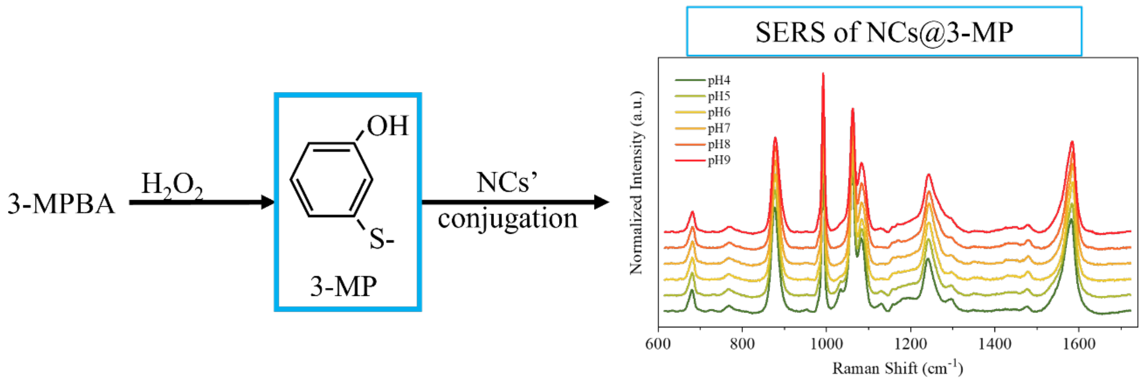

Figure SI-8: SERS spectra of NCs@3-MP at varying pH in the absence of H_2O_2. We took the oxidized form, 3-MP, of the molecular sensor, 3-MPBA, and functionalized the NCs with it. We dispersed the NCs in phosphate buffer at different pH ranging from pH 4 to 9 and measured the SERS response in the absence of H_2O_2.

2.3 The pKa of 3-MPBA determines the sensitivity of boronic acid-based H_2O_2 sensors.

Let's have a closer look to the molecular H_2O_2 sensor, 3-MPBA. An important parameter of 3-MPBA is its pKa value, which is a measure of its Lewis acidity. This value determines the ratio between the trigonal boronic acid and the tetragonal boronate ion (negatively charged) at a specific pH value (figure SI-9A). In the case of NCs@3-MPBA, the inner gold surface will be fully negatively charged when dispersed in a strong basic solution because pKa of phenylboronic acid monolayers is reported to be 9.2[14]. These structural changes of the molecule can be observed using SERS spectroscopy. More specifically, the relative SERS intensity of the 3-MPBA band that is assigned to the non-totally symmetric ring stretching mode (1553 cm^{-1}) varies depending on the environmental pH values, which is a manifestation of charge transfer (CT) processes[15]. Figure SI-9B shows SERS spectra of NCs@3-MPBA dispersed in phosphate buffered saline with pH ranging from 4 to 9. The band at 1553 cm^{-1} decreases in intensity when pH increases. More importantly, pH had relatively low effect on the H_2O_2 sensitive peak at 882 cm^{-1} in the absence of H_2O_2, confirming no crosstalk between the signals and ensuring that changes in the 882 cm^{-1} peak were due to H_2O_2 variations and not to pH changes. 3-MPBA oxidized into 3-MP converting boronate to hydroxyl functional group. This oxidative process produces the rupture of the B-C chemical bond. Comparing with trigonal boronic acid, the complexation with a third hydroxyl group in tetragonal boronate ion facilitates the oxidation to hydroxyl in the presence of H_2O_2, which enhanced this B-C bond cleavage sensitivity and enhances 3-MPBA oxidation by H_2O_2 base[16]. The equilibrium constants of this oxidation reaction vary at specific pH. This can be estimated using the Henderson-Hasselbach equation using the pKa of the 3-MPBA. Under basic pH, lower amount of H_2O_2 is needed than in acid pH to achieve equivalent SERS readout because the reaction is favored. With same amount of H_2O_2, since the equilibrium is different for different pH values, the SERS readout will be different, and thus the limit of detection (LODs) under different pH are going to be also different. The sensitivity of our sensor NCs@3-MPBA is pH-dependent, being maximum at high pH (7-9) and lowers with decreasing pH (6-4). In general, H_2O_2 measurements are based on direct or indirect oxidation of a probe by H_2O_2[5], thus the pH effect on H_2O_2 measurements can be applied to all H_2O_2 sensors which are based on aromatic boronic acid coupled with fluorescence or SERS.

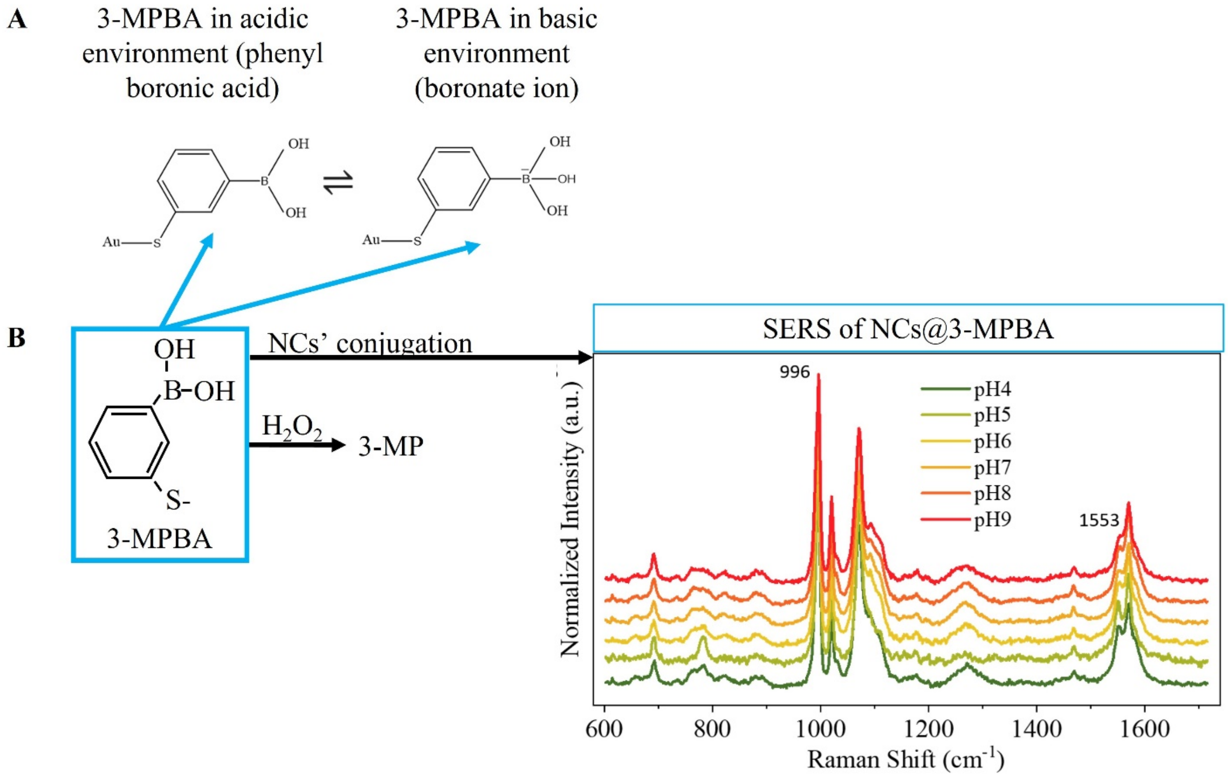

Figure SI-9: SERS spectra of NCs@3-MPBA at varying pH in the absence of H_2O_2. (A) Scheme of 3-MPBA format in acidic pH (phenylboronic acid) and alkaline pH (boronate acid). **(B)** SERS spectra of NCs@3-MPBA dispersed in phosphate buffer with pH ranging from 4 to 9, showing a decreasing intensity at 1553 cm^{-1} (peak ascribed to charge transfer processes) with increasing pH. Each spectrum was the average of 5 spectra obtained from 5 different NCs@3-MPBA.

2.4 NCs@3-MPBA&4-MBA's LOD depends on pH.

Our H_2O_2 sensor's (NCs@3-MPBA&4-MBA) LOD was calculated based on visual definition[17]. We used logarithmic scales for both the horizontal and vertical axes (*i.e.,* log (I_{882}/I_{996}) and log [H_2O_2]), which broaden the linear range to four orders of magnitude (*e.g.,* for pH7 [H_2O_2] from 10^{-2} M to 10^{-6} M), since the linear ranges previous reported were in two orders or less based on numerical scales[1,8,18]. Figure 2 shows the different H_2O_2 calibration curves for each pH and the pH-dependent LODs of the sensor while figure SI-10 shows the corresponding calibration equations. The LOD of H_2O_2 at acid and neutral pH (pH 4 to pH 7) was around 10^{-6} M and close to 10^{-8} M for pH 8 and pH 9. The behavior was like the individual nanosensor (NCs@3-MPBA) (figure SI-11).

Linear fitting: $\log(I_{882}/I_{996}) = a + b*\log[H_2O_2]$					
**pH**	**Linear range (mol/L)**	**a-intercept**	**b-slope**	**R^2**	**LOD**
**4**	10^{-2} to 10^{-4}	-0.134	0.342	0.990	48 μM
**5**	10^{-2} to 10^{-5}	-0.103	0.289	0.969	5.8 μM
**6**	10^{-2} to 10^{-5}	0.052	0.293	0.983	3.9 μM
**7**	10^{-2} to 10^{-6}	0.141	0.254	0.981	0.77 μM
**8**	10^{-2} to 10^{-6}	0.210	0.245	0.961	0.42 μM
**9**	10^{-2} to 10^{-7}	0.229	0.218	0.956	45 nM

Figure SI-10: Calibration curves equations and LODs of NCs@3-MPBA&4-MBA for H_2O_2 sensing at pH ranging from 4 to 9.

A

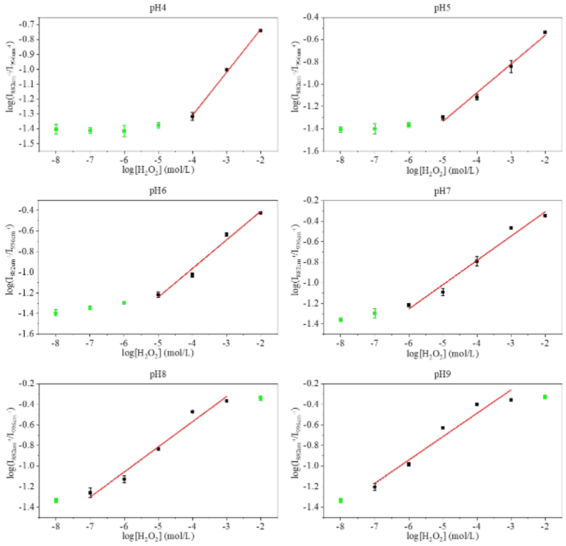

B

pH	Linear range (mol/L)	a-intercept	b-slope	R^2	LOD
	**Linear fitting: $\log(I_{882}/I_{996}) = a + b*\log[H_2O_2]$**				
4	10^{-2} to 10^{-4}	-0.151	0.289	0.997	57 µM
5	10^{-2} to 10^{-5}	-0.046	0.258	0.988	9.5 µM
6	10^{-2} to 10^{-5}	0.146	0.277	0.980	4.6 µM
7	10^{-2} to 10^{-6}	0.163	0.236	0.975	0.76 µM
8	10^{-3} to 10^{-7}	0.409	0.244	0.970	93 nM
9	10^{-3} to 10^{-7}	0.423	0.227	0.951	39 nM

Figure SI-11: NCs@3-MPBA calibration curves and linear ranges for H_2O_2. **(A)** SERS measurements were performed under different pH (from 4 to 9). Red lines are linear fitting results. Green dots were masked (data points not included in the linear fitting). Error bars represented the standard deviations of five probes. **(B)** Calibration curves equations and LODs of NCs@3-MPBA for H_2O_2 sensing in phosphate buffer with pH ranging from 4 to 9.

Section 3: Multiplex nanosensor's cellular internalization and biocompatibility.

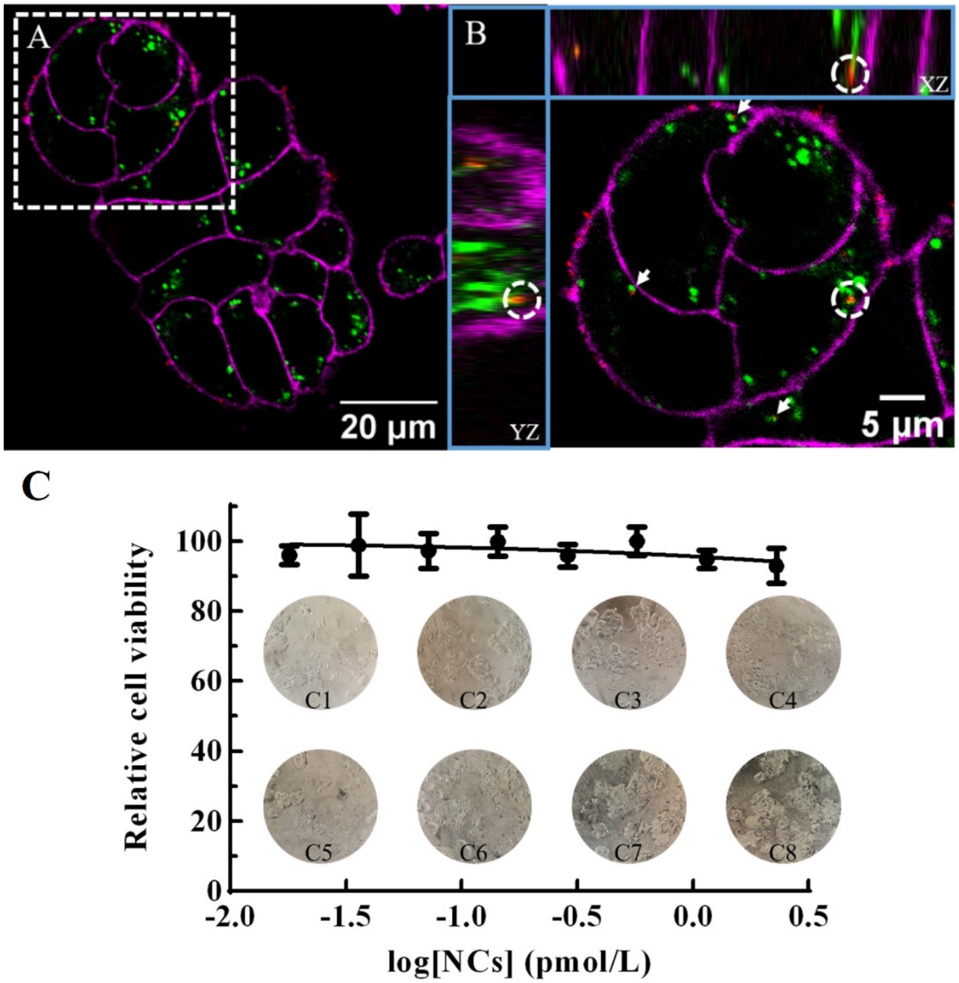

Figure SI-12: NCs cellular internalization and biocompatibility. (A-B) Cellular uptake of NCs by HT29 cells using confocal laser scanning microscopy (CLSM). **(A)** Z-scan of a cells area. **(B)** Different planes (X/Y; X/Z; Y/Z) of selected area (dashed square in A). NCs were shown with white arrow and white dash circle. Internalized NCs were localized within lysosomes as observed by the co-localization of the signal intensities of the different dyes and the reflected light of the NCs. Lysosomes: green; NCs: red; and cells membrane: magenta. **(C)** Cytotoxicity assay of NCs in HT29 cells. Cell viability was determined by Resazurin-Based Assay after the internalization of NCs. The concentrations of NCs were calculated by number. Optical images were collected with OLYMPUS CKX41 inverted microscope. NCs concentration increased from C1 to C8. Since C7 (NCs concentration higher than 1.15 pmol/L), the HT29 cells were fully covered by NCs.

Section 4: Real time and non-invasive multiplexing of pH and H_2O_2 dynamics in living cells.

4.1 Chemical alteration of the cellular H_2O_2 homeostasis to induce oxidative stress.

One of the most common and simply method to understand intracellular H_2O_2 functions is to add H_2O_2 itself directly to the experimental system. To demonstrate the feasibility of mimicking cell stress upon exposure to H_2O_2, we used a fluorescent probe to monitor intracellular changes (figure SI-13A). Figure SI-13A shows cells transfected with orp1-GFP specifically labeling intracellular H_2O_2. After addition of 500 µM H_2O_2 to the cell's growth media, the fluorescence decreases, indicating the presence of intracellular H_2O_2. By calculating integrated density of CLSM images, we observed that the intracellular H_2O_2 level reached a plateau after 10 min treatment (figure SI-13B). We further measure possible cytotoxicity issues that could be derived from the exposure to H_2O_2 to discard erratic cell stress that could affect our sensing. We measured toxicity at the level of mitochondrial activity (figure SI-13C) and cell membrane integrity (figure SI-13D) and confirmed that exposure to H_2O_2 was not cytotoxic and the cells exhibited a cell viability of higher than 90%.

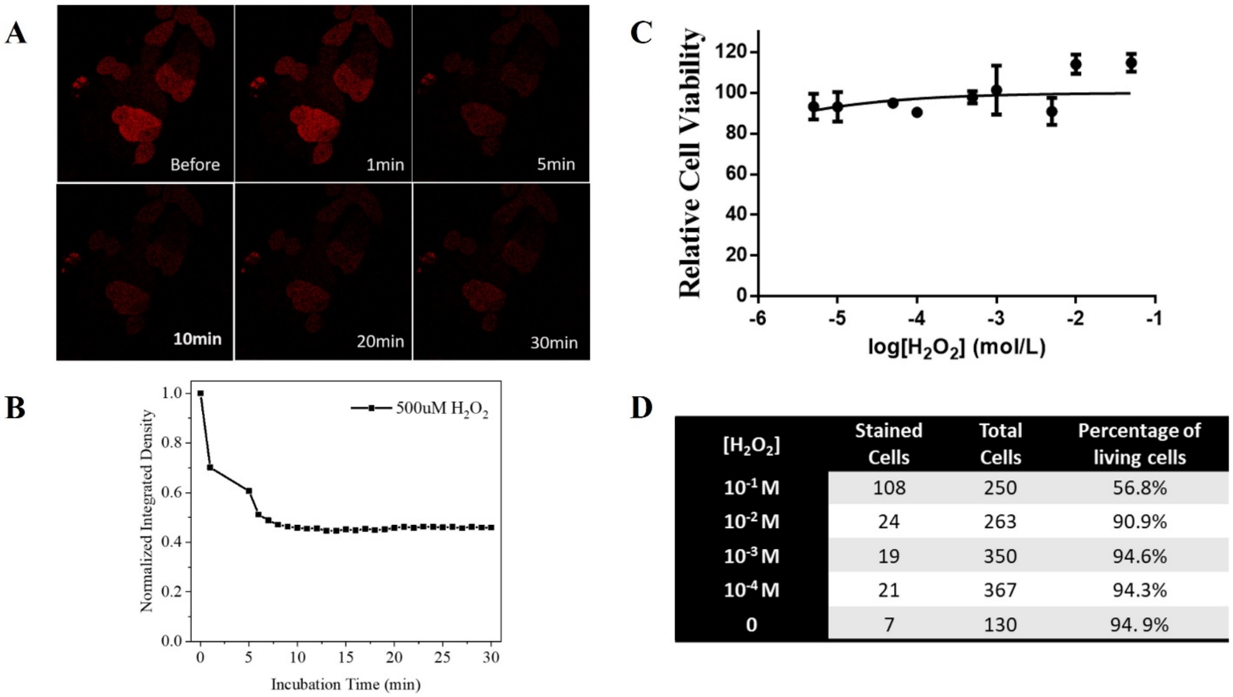

$[H_2O_2]$	Stained Cells	Total Cells	Percentage of living cells
10^{-1} M	108	250	56.8%
10^{-2} M	24	263	90.9%
10^{-3} M	19	350	94.6%
10^{-4} M	21	367	94.3%
0	7	130	94. 9%

Figure SI-13: Altering intracellular H_2O_2 level changes by adding H_2O_2. (A) CLSM images of orp1-GFP transfected HT29 (MOI 80): before and after 0.5 mM H_2O_2 addition 1 min, 5 min, 10 min, 20 min and 30 min. **(B)** Integrated density calculated with ImageJ before and after 0.5 mM H_2O_2 addition. The decrease went into a platform after 10 min. **(C)** Resazurin-based viability assay after 30 min of H_2O_2. H_2O_2 concentration $5x10^{-2}$ M, 10^{-2} M, $5x10^{-3}$ M, 10^{-3} M, $5x10^{-4}$ M, 10^{-4} M, $5x10^{-5}$ M, 10^{-5} M, $5x10^{-6}$ M. Mitochondria activity increased under H_2O_2 stimulation. **(D)** Trypan blue viability assay of H_2O_2 treated HT29 to check cell membrane integrity.

4.2 Inducing intracellular alkalinization to alter pH homeostasis.

Bafilomycin A1 is one commonly used agent modifying lysosomal acidification[10]. The vacuolar ATPase (V-ATPase) is a proton pump hydrolysing ATP, controlling the acidification of endosomes and lysosomes. Bafilomycin A1 could inhibit the activity of the V-ATPase[19,20,21]. Thus, lysosomal pH increase upon the

addition of Bafilomycin A1[20]. We also checked the ability in our cell line by using lysotracker as a fluorescence pH indicator shown in figure SI-14. With Bafilomycin A1 500 nM treated after 2 hours, there was no signals coming from lysotracker, which meant the lysosomal pH was not acidic.

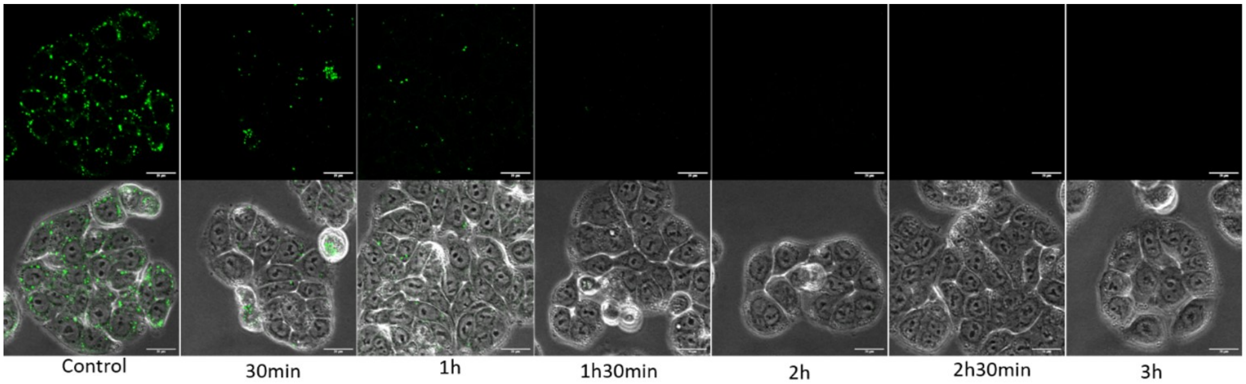

Figure SI-14: Altering pH homeostasis with bafilomycin A1. Cells HT29 were treated with 500 nM Bafilomycin A1 over time. Endolysosomal pH changes are visualized with lysotracker (green).

4.3 Cellular H_2O_2 sensing with NCs@3-MPBA.

Figure SI-15 shows the intracellular and extracellular NCs@3-MPBA SERS spectra collected with untreated HT29 and H_2O_2 treated HT29 cells. By comparing intensity ratio $\log(I_{882}/I_{996})$ of untreated cells and H_2O_2 treated cells (10 mM, 1 mM, and 0.5 mM), we observed that both the intracellular and extracellular H_2O_2 level increased when H_2O_2 concentration increased, indicating the sensing ability of our sensor. Meanwhile, the intracellular signal was much lower than the extracellular signal.

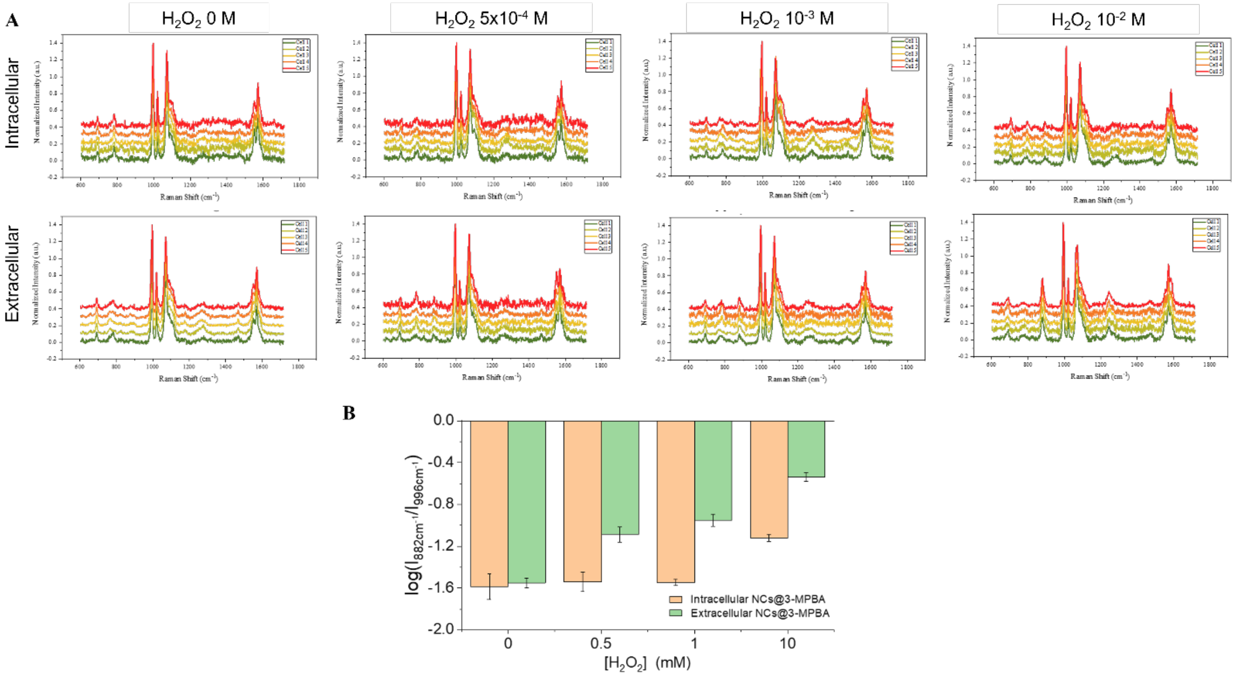

Figure SI-15: SERS spectra of intracellular and extracellular NC@3-MPBA of HT29 treated with different amount of H_2O_2 (10 mM, 1 mM, 0.5 mM, 0mM). Each spectrum was collected with a NCs@3-MPBA from 5 different cells. **(A)** raw data and **(B)** average values of the different NCs.

4.4 Cellular multiplexing of H_2O_2 and pH imbalances with NCs@3-MPBA&4-MBA.

Figure 4 shows the distribution of I_{1385}/I_{996} and $\log(I_{882}/I_{996})$ obtained from individual NCs' spectra corresponding to the different pH and H_2O_2 values measured for all samples whereas figures 4C and 4D show the average value of different experiments and the standard deviation of I_{1385}/I_{996} (pH) and $\log(I_{882}/I_{996})$ (H_2O_2) separately. By knowing the intensity ratio of I_{1385}/I_{996} and $\log(I_{882}/I_{996})$ (figure 4B-4D) and relating them to the pH (*cf.,* SI§1, figure SI-6A) and H_2O_2 calibration curves (figure 2 and *cf.,* SI§2, figure SI-10), we can estimate the pH and H_2O_2 values for all samples.

Figure SI-16 shows the complete SERS spectra from which the zoomed are shown in figure 4A has been taken.

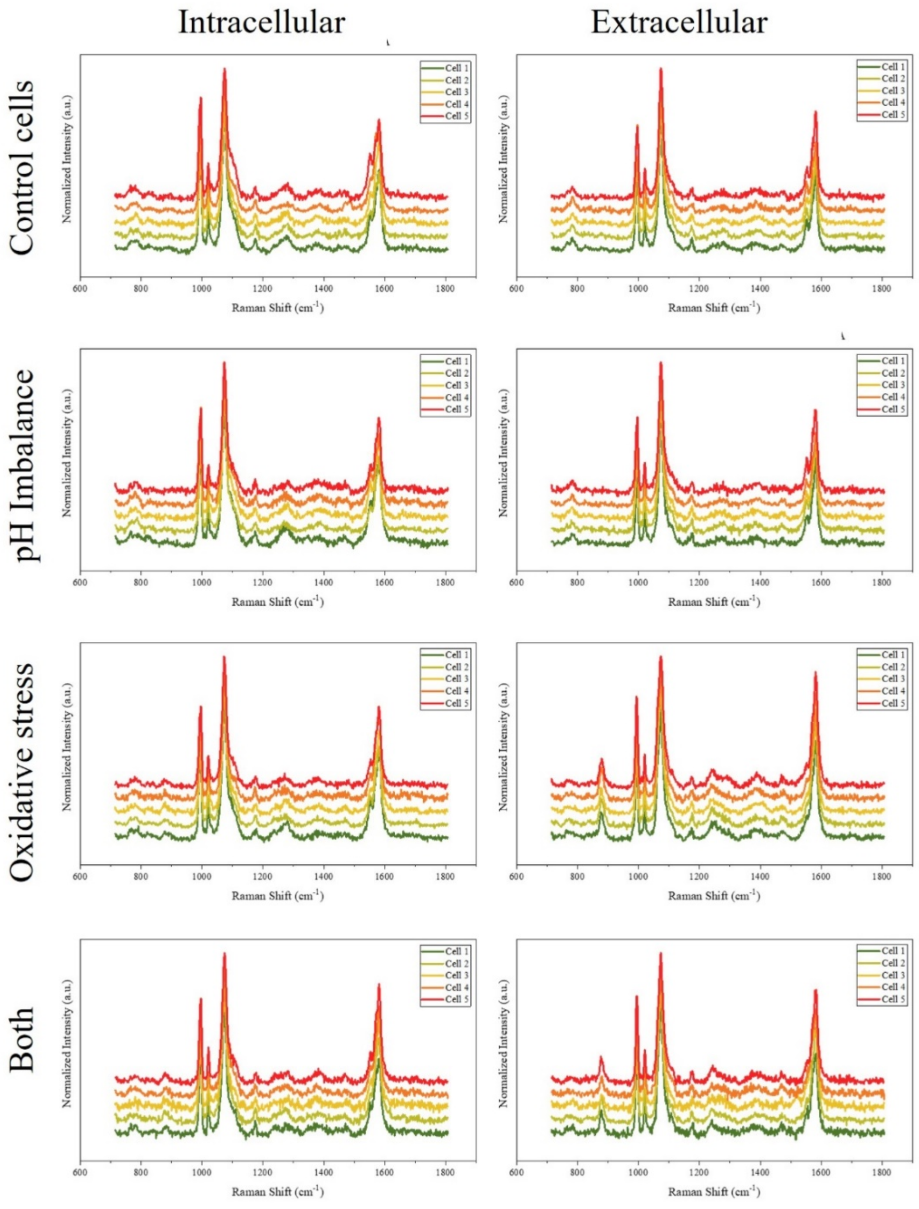

Figure SI-16: NCs@3-MPBA&4-MBA real time, live cells H_2O_2 and pH SERS determination. Intracellular and extracellular SERS spectra of untreated (control) and treated HT29 cell samples. The treatments were: Bafilomycin A1 treated, 10 mM H_2O_2, and Bafilomycin A1 and 10 mM H_2O_2. Each spectrum was collected with one NCs@3-MPBA&4-MBA in 5 different cells.

	C-I		C-E		B-I		B-E		H-I		H-E		BH-I		BH-E	
	pH	H_2O_2	pH	H_2O_2	pH	H_2O_2	pH	H_2O_2	pH	H_2O_2	pH	H_2O_2	pH	H_2O_2	pH	H_2O_2
**Cell 1**	6	<4E-6	7	<8E-7	7	<8E-7	7	<8E-7	6	6E-05	7	2E-03	7	1E-05	7	1E-03
**Cell 2**	6	<4E-6	7	1E-06	7	<8E-7	7	<8E-7	6	6E-05	7	2E-03	7	5E-05	7	2E-03
**Cell 3**	6-7	<4E-6	7	<8E-7	7	<8E-7	7	<8E-7	6-7	2E-05	7	2E-03	7	4E-05	7	2E-04
**Cell 4**	6-7	<4E-6	7	<8E-7	7	<8E-7	7	<8E-7	6-7	1E-05	7	3E-03	7	2E-05	7	8E-04
**Cell 5**	6-7	<4E-6	7	1E-06	7	<8E-7	7	<8E-7	6-7	1E-05	7	2E-03	7	3E-05	7	1E-03

Table SI-2: Intracellular and extracellular pH and H_2O_2 concentration calculated based on calibration curve of all the NCs@3-MPBA&4-MBA measured. Intracellular and extracellular SERS spectra were collected with HT29 under different treatments. C-I: intracellular probes of blank HT29; C-E: extracellular probes of blank HT29; B-I: intracellular probes of Bafilomycin A1 treated HT29; B-E: extracellular probes of Bafilomycin A1 treated HT29; H-I: intracellular probes of 10 mM H_2O_2 treated HT29; H-E: extracellular probes of 10 mM H_2O_2 treated HT29; BH-I: intracellular probes of Bafilomycin A1 and 10 mM H_2O_2 treated HT29; BH-E: extracellular probes of Bafilomycin A1 and 10 mM H_2O_2 treated HT29.

4.5 High irradiation time causes photosublimation of 4-MBA which results in an altered spectrum.

SERS measurements in biological environment such as a cell is complex due to many interferences[22] this cause that the irradiation parameters set up for cells are more extreme than in a buffer. For example, irradiation time of the NCs is higher due to the scattering from the cellular structures and the difficulty to focus as much as light as possible to obtain enough SERS intensity. We observed that increasing the irradiation time affects the stability of the molecular sensor. We took NCs@3-MPBA&4-MBA functionalized at a ratio 5:1 and dispersed in a phosphate saline buffer at pH 9 without H_2O_2. Figure SI-17 shows the SERS response after their irradiation of the same NC at a 5 mW laser power during 5 s and 20 s. The intensities of peaks at 1385 cm^{-1} and 1590 cm^{-1}, corresponding to symmetric carboxyl stretching mode and aromatic ring vibrations of 4-MBA, decreased after 20 s. Possibly, high irradiation times induce long-lasting increase in the local temperature of the plasmonic nanostructure (Au) where 4-MBA is conjugated. An increased and continuous heating of 4-MBA after laser irradiation can result in a photo-induced sublimation[23] of 4-MBA. We observed this effect on 4-MBA but not on 3-MPBA at the irradiation times described. Thus, the photosensitivity of the Raman probes is different most probably because of their different molecular structure. We can conclude that the irradiation time must be check when selecting a Raman probe for biosensing as it may interfere with the quantification.

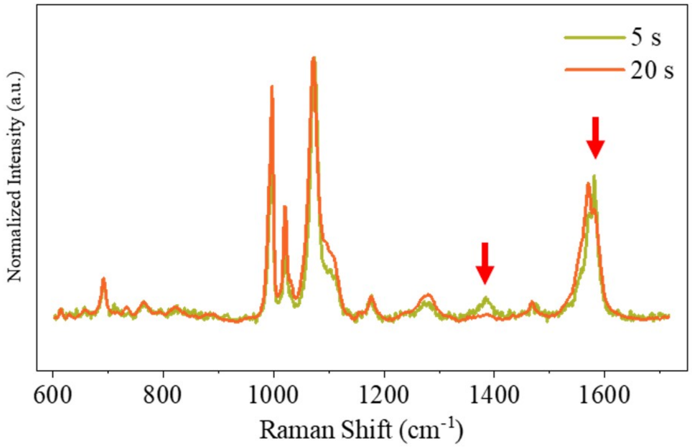

Figure SI-17: SERS spectra of NCs@3-MPBA&4-MBA: The NCs were functionalized at a 5:1 ratio ($5x10^{-3}$ M 3-MPBA : 10^{-3} M 4-MBA) and dispersed in a phosphate buffered saline at pH 9. Individual NCs were irradiated for 5 s and 20 s at a fixed laser power (5 mW) and the spectra were collected. Red arrows indicate affected peaks at 1385 cm^{-1} and 1590 cm^{-1} corresponding to 4-MBA.

References

(1) Gu, X.; Wang, H.; Schultz, Z. D.; Camden, J. P. Sensing Glucose in Urine and Serum and Hydrogen Peroxide in Living Cells by Use of a Novel Boronate Nanoprobe Based on Surface-Enhanced Raman Spectroscopy. *Anal. Chem.* **2016**, *88* (14), 7191–7197.

(2) Lee, H. M.; Kim, M. S.; Kim, K. Surface-Enhanced Raman Scattering of Ortho- and Para-Mercaptophenols in Silver Sol. *Vib. Spectrosc.* **1994**, *6*, 205–214.

(3) Dickinson, B. C.; Huynh, C.; Chang, C. J. A Palette of Fluorescent Probes with Varying Emission Colors for Imaging Hydrogen Peroxide Signaling in Living Cells. *J. Am. Chem. Soc.* **2010**, *132* (16), 5906–5915.

(4) Duellman, S., Shultz, J., Vidugiris, G., and Cali, J. A New Luminescent Assay for Detection of Reactive Oxygen Species https://www.promega.es/en/resources/pubhub/a-luminescent-assay-for-detection-of-reactive-oxygen-species/ (accessed Aug 10, 2022).

(5) Rezende, F.; Brandes, R. P.; Schröder, K. Detection of Hydrogen Peroxide with Fluorescent Dyes. *Antioxidants Redox Signal.* **2018**, *29* (6), 585–602.

(6) Belousov, V. V.; Fradkov, A. F.; Lukyanov, K. A.; Staroverov, D. B.; Shakhbazov, K. S.; Terskikh, A. V.; Lukyanov, S. Genetically Encoded Fluorescent Indicator for Intracellular Hydrogen Peroxide. *Nat. Methods* **2006**, *3* (4), 281–286.

(7) Gutscher, M.; Sobotta, M. C.; Wabnitz, G. H.; Ballikaya, S.; Meyer, A. J.; Samstag, Y.; Dick, T. P. Proximity-Based Protein Thiol Oxidation by H2O2-Scavenging Peroxidases. *J. Biol. Chem.* **2009**, *284* (46), 31532–31540.

(8) Li, X.; Duan, X.; Yang, P.; Li, L.; Tang, B. Accurate in Situ Monitoring of Mitochondrial H2O2 by Robust SERS Nanoprobes with a Au−Se Interface. *Anal. Chem.* **2021**, *93* (8), 4059–4065.

(9) Pazos, E.; Garcia-Algar, M.; Penas, C.; Nazarenus, M.; Torruella, A.; Pazos-Perez, N.; Guerrini, L.; Vázquez, M. E.; Garcia-Rico, E.; Mascareñas, J. L.; Alvarez-Puebla, R. A. Surface-Enhanced Raman Scattering Surface Selection Rules for the Proteomic Liquid Biopsy in Real Samples: Efficient Detection of the Oncoprotein c-MYC. *J. Am. Chem. Soc.* **2016**, *138* (43), 14206–14209.

(10) Kneipp, J.; Kneipp, H.; Wittig, B.; Kneipp, K. One- and Two-Photon Excited Optical PH Probing for Cells Using Surface-Enhanced Raman and Hyper-Raman Nanosensors. *Nano Lett.* **2007**, *7* (9), 2819–2823.

(11) García-Algar, M.; Tsoutsi, D.; Sanles-Sobrido, M.; Cabot, A.; Izquierdo-Roca, V.; Gil, H. P. R. Subcellular Optical PH Nanoscale Sensor. *ChemistrySelect* **2017**, *2* (26), 8115–8121.

(12) Michota, A.; Bukowska, J. Surface-Enhanced Raman Scattering (SERS) of 4-Mercaptobenzoic Acid on Silver and Gold Substrates. *J. Raman Spectrosc.* **2003**, *34* (1), 21–25.

(13) Radić, N.; Prkic, A. Historical Remarks on the Henderson-Hasselbalch Equation: Its Advantages and Limitations and a Novel Approach for Exact PH Calculation in Buffer Region. *Rev. Anal. Chem.* **2012**, *31* (2), 93–98.

(14) Takahashi, S.; Anzai, J. I. Phenylboronic Acid Monolayer-Modified Electrodes Sensitive to Sugars. *Langmuir* **2005**, *21* (11), 5102–5107.

(15) Osawa, M.; Matsuda, N.; Yoshii, K.; Uchida, I. Charge Transfer Resonance Raman Process in Surface-Enhanced Raman Scattering from p-Aminothiophenol Adsorbed on Silver: Herzberg-Teller Contribution. *J. Phys. Chem.* **1994**, *98* (48), 12702–12707.

(16) Barriet, D.; Yam, C. M.; Shmakova, O. E.; Jamison, A. C.; Lee, T. R. 4-Mercaptophenylboronic Acid SAMs on Gold: Comparison with SAMs Derived from Thiophenol, 4-Mercaptophenol, and 4-Mercaptobenzoic Acid. *Langmuir* **2007**, *23* (17), 8866–8875.

(17) Şengül, Ü. Comparing Determination Methods of Detection and Quantification Limits for Aflatoxin Analysis in Hazelnut. *J. Food Drug Anal.* **2016**, *24* (1), 56–62.

(18) Zhang, C.; Liu, X.; Xu, Z.; Liu, D. Multichannel Stimulus-Responsive Nanoprobes for H2O2 Sensing in Diverse Biological Milieus. *Anal. Chem.* **2020**, *92* (18), 12639–12646.

(19) De Luca, M.; Ferraro, M. M.; Hartmann, R.; Rivera-Gil, P.; Klingl, A.; Nazarenus, M.; Ramirez, A.; Parak, W. J.; Bucci, C.; Rinaldi, R.; Del Mercato, L. L. Advances in Use of Capsule-Based

Fluorescent Sensors for Measuring Acidification of Endocytic Compartments in Cells with Altered Expression of V-ATPase Subunit V1G1. *ACS Appl. Mater. Interfaces* **2015**, *7* (27), 15052–15060.
(20) Rivera-Gil, P.; Nazarenus, M.; Ashraf, S.; Parak, W. J. PH-Sensitive Capsules as Intracellular Optical Reporters for Monitoring Lysosomal PH Changes upon Stimulation. *Small* **2012**, *8* (6), 943–948.
(21) Kastl, L.; Sasse, D.; Wulf, V.; Hartmann, R.; Mircheski, J.; Ranke, C.; Carregal-Romero, S.; Martínez-Ló, J. A.; Ferná Ndez-Chacó, R.; Parak, W. J.; Elsasser, H.-P. P.; Rivera_Gil, P.; Martinez-Lopez, J. A.; Fernandez-Chacon, R.; Parak, W. J.; Elsasser, H.-P. P.; Rivera Gil, P. Multiple Internalization Pathways of Polyelectrolyte Multilayer Capsules into Mammalian Cells. *ACS Nano* **2013**, *7* (8), 6605–6618.
(22) Tsoutsi, D.; Sanles-Sobrido, M.; Cabot, A.; Gil, P.-R. Common Aspects Influencing the Translocation of SERS to Biomedicine. *Curr. Med. Chem.* **2018**, *25* (35), 4638–4652.
(23) Álvarez-Puebla, R. A. Effects of the Excitation Wavelength on the SERS Spectrum. *J. Phys. Chem. Lett.* **2012**, *3* (7), 857–866.
